# Assessing the Consequences of Denoising Marker-Based Metagenomic Data

**DOI:** 10.1371/journal.pone.0060458

**Published:** 2013-03-25

**Authors:** John M. Gaspar, W. Kelley Thomas

**Affiliations:** Department of Molecular, Cellular, & Biomedical Sciences, University of New Hampshire, Durham, New Hampshire, United States of America; Argonne National Laboratory, United States Of America

## Abstract

Early marker-based metagenomic studies were performed without properly accounting for the effects of noise (sequencing errors, PCR single-base errors, and PCR chimeras). Denoising algorithms have been developed, but they were validated using data derived from mock communities, in which the true sequences were known. Since the algorithms were designed to be used in real community studies, it is important to evaluate the results in such cases. With this goal in mind, we processed a real 16S rRNA metagenomic dataset through five denoising pipelines. By reconstituting the sequence reads at each stage of the pipelines, we determined how the reads were being altered. In one denoising pipeline, AmpliconNoise, we found that the algorithm that was designed to remove pyrosequencing errors changed the reads in a manner inconsistent with the known spectrum of these errors, until one of the parameters was increased substantially from its default value. Additionally, because the longest read was picked as the representative for each cluster, sequences were added to the 3′ ends of shorter reads that were often dissimilar from what had been removed by the truncations of the previous filtering step. In QIIME, the denoising algorithm caused a much larger number of changes to the reads unless the parameters were changed from their defaults. The denoising pipeline in mothur avoided some of these negative side-effects because of its strict default filtering criteria, but these criteria also greatly limited the sequence information produced at the end of the pipeline. We recommend that those using these denoising pipelines be cognizant of these issues and examine how their reads are being transformed by the denoising process as a component of their analysis.

## Introduction

The emerging field of metagenomics is concerned with determining the numbers and types of organisms in a particular environment. Studies of microbes (the “microbiome”) have principally focused on sequence analysis of the 16S ribosomal RNA gene [1], which contains nine variable regions flanked by sequences that are conserved across most bacterial species. The advent of PCR, followed by new sequencing technologies such as pyrosequencing, have offered us the potential to reach deep into the rare biosphere. A single PCR reaction of DNA extracted from a given sample can yield more than a million sequence reads in a pyrosequencing run. While many of these reads may be easily identified as belonging to well-studied species [2], [3], some may not match any sequences in the database, simply because the bacteria have not been cultured or otherwise classified. Those reads may represent new species, or they may be rare variants of a known species that differ from the database sequence by just a few nucleotides. Therefore, it becomes necessary to cluster the reads to a certain percent identity (generally, 3% for species-level clustering) to determine how many types of bacteria, or operational taxonomic units (OTUs), are present in a given sample.

Recent metagenomic studies have shown a much larger diversity of prokaryotic species than previously thought, in samples ranging from the human gut [4] to the deep sea [5]. These studies have since been questioned in light of the realization that artifacts can result in the overestimation of OTU numbers. These artifacts include sequencing errors, PCR single-base errors, and PCR chimeras [6].

Pyrosequencing, like Sanger sequencing, has a low but nonzero error rate. In Roche-454 pyrosequencing, errors arise due to the inaccurate determination of homopolymer lengths [7]–[9]. Studies have been performed to analyze ways in which to improve the accuracy of reads by using different filtering criteria [10]. However, in order to prevent an overestimation of OTU number, additional criteria must be used, resulting in the removal of a large percentage of reads [11].

In addition to pyrosequencing errors, PCR itself can introduce noise. The polymerases used in PCR, like all polymerases, do not replicate DNA perfectly. Also, when performing multi-template PCR in a metagenomic study, the possibility exists for forming chimeras. A chimera is a PCR product that is composed of parts of two (or more) different templates, thought to result when the product of an incomplete extension in one cycle mis-primes on a different template in a subsequent cycle of the reaction.

To limit the effects of noise in marker-based metagenomic analyses, “denoising” tools have been developed. One early algorithm for removing noise was PyroNoise [6]. The principal advance with this program was that it focused on the flowgrams – the raw pyrosequencing data from the Roche-454 platform – instead of the DNA sequences. In this way, pyrosequencing errors could be modeled more appropriately. PyroNoise used an iterative expectation-maximization algorithm to cluster the flowgrams, followed by an adaptation of the Mallard algorithm to screen for PCR chimeras.

Recently, Quince et al. [12] demonstrated the use of an improvement of this pipeline called AmpliconNoise. In AmpliconNoise, following the initial filtering steps, pyrosequencing errors are removed by flowgram clustering (by an algorithm also called “PyroNoise”), but this is done without alignment, unlike the original PyroNoise. After the sequences are truncated, another algorithm, SeqNoise, removes PCR single-base errors by further clustering the reads according to a maximum likelihood model that incorporates PCR error rates. Finally, an algorithm called Perseus is used to remove PCR chimeras.

AmpliconNoise was benchmarked on datasets generated using known sets of reference templates (“mock communities”). The sequential application of the algorithms of AmpliconNoise allowed the correct OTU number at various levels of sequence difference to be determined, and the reads after AmpliconNoise had decreased error rates compared to the raw reads. Additionally, by separating the steps of denoising, the run-time of the entire set of algorithms was greatly reduced compared to the original PyroNoise.

Related denoising algorithms have been developed in the microbial ecology analysis packages QIIME [13] and mothur [14]. In QIIME, the denoising pipeline was designed as a faster version of the original PyroNoise [15]. The algorithm denoiser aligns and clusters flowgrams in a single step, so as to take into account both pyrosequencing errors and PCR single-base errors. Following this, the reads can be checked for chimeras by ChimeraSlayer [16] or blast_fragments.

In mothur, the PyroNoise and SeqNoise algorithms of AmpliconNoise have been recoded as shhh.flows and shhh.seqs [17]. Chimera removal is accomplished by rewritten versions of Perseus, ChimeraSlayer, or UCHIME [18]. These algorithms were analyzed, along with various filtering and trimming programs available in mothur, to produce a pipeline that minimized the error rates while maintaining as many reads as possible.

Other denoising algorithms have been developed that do not incorporate the analysis of flowgrams. Single-linkage preclustering (SLP) [19] is based on the notion that reads containing errors should be distributed around more abundant error-free reads. Therefore, by preclustering those reads, one can produce OTUs using just the error-free reads, thus reducing the number of erroneous OTUs. Another algorithm, Acacia [20], has recently been shown to produce results similar to those of AmpliconNoise and the QIIME denoising pipeline while using a fraction of the computational time.

**Figure 1:**
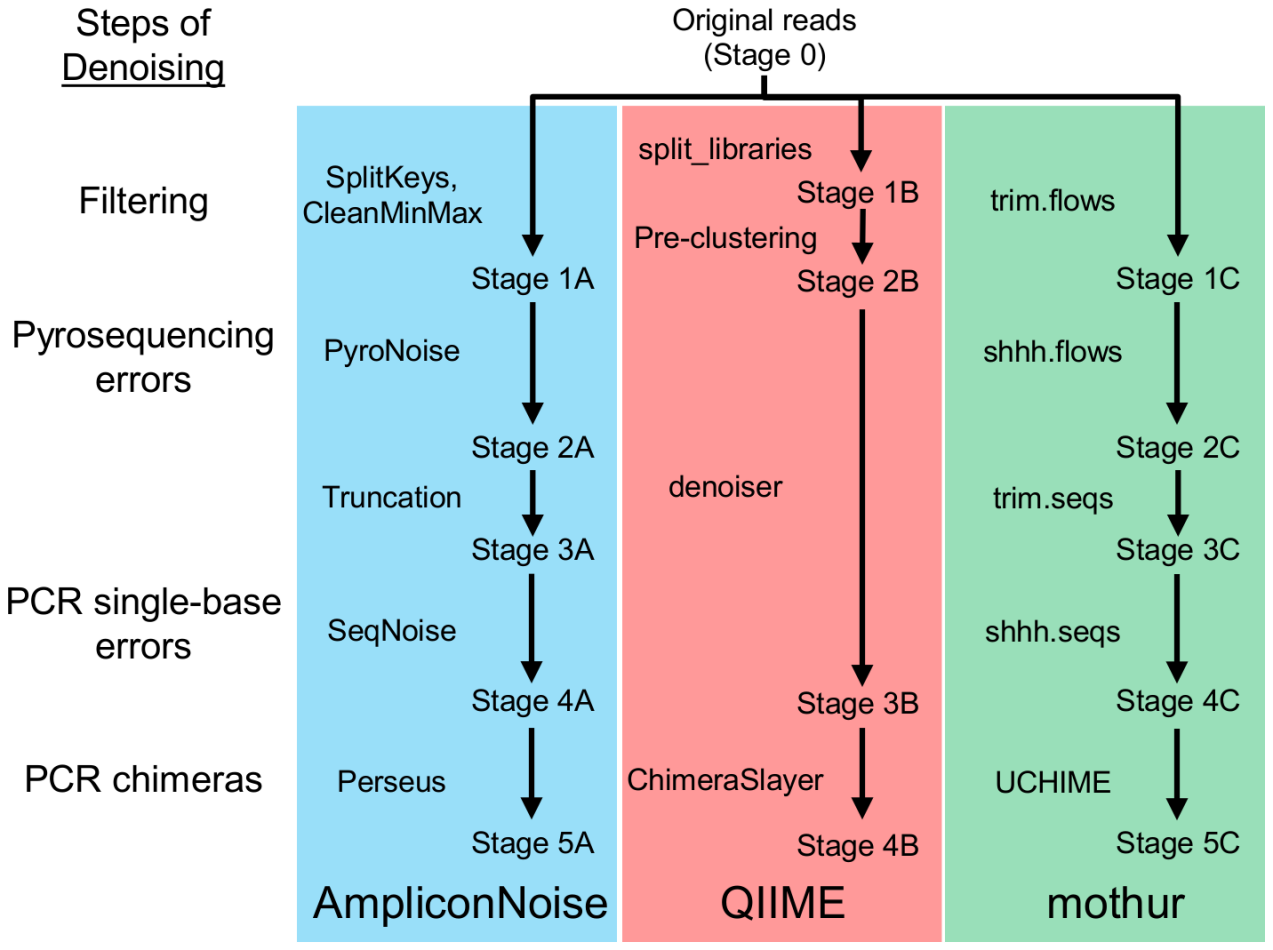
The steps of three denoising pipelines. The reads were reconstituted at each stage following the steps, as described in the text. The reads will be referred to by their stage numbers throughout this paper.

Each of these denoising pipelines was validated and optimized using mock community data, in which the true sequences and the correct OTU number were known. However, they were designed to be used in studies of real communities, so it is important to evaluate their results in such cases. Here we propose methods by which this can be accomplished. We break down each pipeline into its component steps and reconstitute the sequence reads at each stage (Fig. 1). Using this approach, we can determine what changes have been made to the individual reads and whether those changes are consistent with removing errors. If they are not, we can adjust the parameters of the algorithms accordingly. The changes are recorded in four categories: substitutions, insertions, deletions, and “3′ gap,” which is the number of bases removed (or added) to the 3′ end of a read.

## Results and Discussion

For this study, we derived a metagenomic dataset from fourteen individual nematodes that were selected from ocean sediment samples taken from off the New Hampshire coast and the Gulf of Mexico. Genomic DNA from these samples, which included the DNA from any associated microbes, was used as the template in PCR amplifications of two regions of the bacterial 16S ribosomal RNA gene (V3–V5 and V6–V8). These amplicons were pyrosequenced using the Roche-454 GS FLX platform with the Titanium protocol (800 flows), resulting in more than 40,000 reads. These reads were processed through each of the pipelines (Fig. 1).

### AmpliconNoise

One of the most popular denoising pipelines is AmpliconNoise [12], which consists of five steps (Fig. 1).

#### Filtering

The first step of AmpliconNoise is performed by the script FlowsMinMax.pl for Titanium data. With our multiplex data, it was easier to divide this step into its two component parts: SplitKeys.pl and CleanMinMax.pl.

The application of SplitKeys.pl to our data resulted in the extraction of 40,596 flowgrams from the sff.txt file into 56 different bins (one for each combination of four PCR primers and fourteen samples). Although it has been shown that reads with two or fewer errors in the primer sequences do not contain significantly more errors in the rest of the read [10], [17], SplitKeys.pl required an exact match of the mid tag - primer sequences. Allowing just a single mismatch or in/del with our data would have resulted in an additional 2,382 reads, an increase of 5.9%.

Next, CleanMinMax.pl filtered our data according to the following process. Each flowgram was analyzed four flows at a time (T – A – C – G; see File S1). It was truncated prior to a set of flows if any of the following was true: (1) all four flow values were less than 0.50; (2) any of the four was greater than 6.49 (in FlowsMinMax.pl, this value is 9.49); (3) any of the four was greater than 0.50 and less than 0.70. Following any truncation, the read was thrown out if fewer than 360 flows remained. If more than 720 flows remained, the flowgram was truncated to 720.

As a result of CleanMinMax, 21.5% of the reads retrieved by SplitKeys were eliminated (Table 1). Most of these eliminations were due to a single flow value between 0.50 and 0.70 (which occurred somewhere in a read’s first 360 flows), and more than 80% of the remaining reads were truncated due to the same criterion. This criterion was justified in the AmpliconNoise paper [12] by referencing a previous study. However, that study [10] did not conclude that a single flow value in that range would place all subsequent flows in question; it simply cited documentation from 454 that stated that reads should be limited to having less than 3% of flows with those values.

**Table 1:** Results of CleanMinMax in the Filtering step of AmpliconNoise.

	Too few flows (< 357)	No signal for four flows	Large flow value (> 6.49)	Flow value between 0.50 and 0.70	Too many flows (> 720)	Total number
Reads eliminated	3032	8	185	5503	N/A	8728
Reads truncated	N/A	268	40	17734	7660	25702

40,596 flowgrams were retrieved by SplitKeys.pl and analyzed by CleanMinMax.pl.

After CleanMinMax, we used the AmpliconNoise script ConvertDatFasta.pl to interpret DNA sequences from the cleaned flowgrams. We then determined what alterations had been made to each of the reads by comparing them (Stage 1A) to the original reads (Stage 0) using our script AlignClusMus.pl.

As shown in Table 2, the 3′ gap was negative due to the truncations of CleanMinMax, which removed an average of 70.4 base pairs from the ends of the reads, up to a maximum of 311 bp. Additionally, there were a total of 1,233 changes – substitutions, insertions, and deletions – in the reads. These were due to differences in the interpretation of flowgrams by ConvertDatFasta, compared to that of the 454 software. All flow values that ended in “.50” were rounded up by ConvertDatFasta, whereas these flow values were rounded down by the 454 software a majority of the time (data not shown). This discrepancy accounted for most of the insertions. Most of the deletions were of ambiguous bases (Ns) in the original reads. Although CleanMinMax was designed to truncate the flowgrams prior to any ambiguous base (criterion (1) above), it missed many ambiguous bases because it analyzed the flow values four at a time and in only one frame (File S1).

**Table 2:** Changes made to the reads at each step of the denoising pipelines.

Denoising Pipeline	Step	Reference reads (stage)	Query reads (stage)	Number of reads analyzed	Mean 3′ gap (S.D.)	Substitutions	Insertions	Deletions	Total changes
AmpliconNoise									
	Filtering	0	1A	31868	–70.4 (75.6)	110	675	448	1233
	PyroNoise	1A	2A	31868	69.2 (82.7)	5571	4003	4073	13647
	Accordion effect	0	2A	31868	–1.1 (69.2)	11177	7754	9814	28745
	Truncation	2A	3A	31868	–36.7 (22.1)	0	0	0	0
	SeqNoise	3A	4A	31868	–4.6 (51.3)	17395	1425	1630	20450
	Perseus	4A	5A	31415	0.0 (0.0)	0	0	0	0
	Net results	0	5A	31415	–42.2 (71.9)	26924	6008	9627	42559
CleanOpt (trunc)		0	1A	31693	–71.1 (75.2)	0	0	475	475
	PyroNoise	1A	2A	31693	68.9 (82.6)	5331	3901	3485	12717
	Accordion effect	0	2A	31693	–2.1 (69.1)	11073	7627	9428	28128
CleanOpt (no trunc)		0	1A	37495	0.0 (0.1)	135	16	1538	1689
	PyroNoise	1A	2A	37495	37.8 (63.7)	10152	11149	13340	34641
	Accordion effect	0	2A	37495	37.8 (63.7)	10166	10187	13897	34250
QIIME									
	split_libraries	0	1B	36281	–48.1 (57.8)	0	0	0	0
	Pre-clustering	1B	2B	36281	70.9 (74.4)	95	600	406	1101
	Accordion effect	0	2B	36281	22.8 (54.8)	226	1384	902	2512
	denoiser	2B	3B	36281	30.0 (60.8)	87925	32869	36449	157243
	ChimeraSlayer	3B	4B	35946	0.0 (0.0)	0	0	0	0
	Net results	0	4B	35946	52.9 (75.0)	86104	31278	33813	151195
mothur									
	trim.flows	0	1C	25119	–167.4 (44.8)	24	230	145	399
	shhh.flows	1C	2C	25119	0.0 (0.1)	2168	897	1283	4348
	Accordion effect	0	2C	25119	–167.4 (44.8)	2360	921	1236	4517
	trim.seqs	2C	3C	25119	0.0 (0.0)	0	0	0	0
	shhh.seqs	3C	4C	25119	–0.1 (1.6)	5306	210	345	5861
	chimera.uchime	4C	5C	24782	0.0 (0.0)	0	0	0	0
	Net results	0	5C	24782	–167.5 (44.9)	7644	1071	1542	10257
SLP									
	split_libraries	0	1D	35166	–18.8 (23.8)	0	0	0	0
	slp.pl	1D	2D	35166	9.2 (66.0)	62571	13743*	18893*	95207
	Net results	0	2D	35166	–9.6 (72.7)	62599	13743*	19040*	95382
Acacia									
	Net results	0	1E	37802	–6.6 (29.1)	7760	4490	7920	20170

All data are in units of base pairs, except those indicated by * (units of events). Rank-abundance curves can be found in Files S25 and S26.

#### PyroNoise

The goal of this step is to correct pyrosequencing errors by clustering the flowgrams that passed the Filtering step. PyroNoise takes two parameters. The parameter -c is the cut-off used to form clusters to initialize the expectation-maximization algorithm, and it has a default value of 0.01. The parameter -s is the inverse of the characteristic cluster size used in the calculations of the expectation step, and it has a default value of 60.0 [12].

We applied PyroNoise to our data using the default values. This resulted in 9,192 clusters of varying sizes. Of those clusters, 75.5% consisted of a single read, although this amounted to just 21.8% of the total number of reads. The largest cluster contained 2,604 reads. Each cluster had a set of reads that mapped to it, along with a single sequence. Since all subsequent steps of the pipeline were performed on the cluster sequences only, we used these sequences to represent all of the reads in their respective clusters (File S2). We then determined what changes had been made to the reads (Stage 2A), using the Stage 1A reads as the reference.

Unlike the Filtering step, PyroNoise produced reads that either stayed the same length or got longer, with an average 3′ gap of +69.2 bp (Table 2). This was due to the fact that, in all but five clusters, the sequence for a given cluster was determined by its longest read. Therefore, instead of forming a consensus, PyroNoise selected a single read to be the representative for each cluster. If a representative read had a difference at one position with some of the other reads in its cluster – or even all of the other reads (File S3) – that difference was spread to those reads. This occurred even if the difference were legitimate, such as a polymorphism, or if the representative read actually had an error at that position, due to PCR, pyrosequencing, or even flowgram misinterpretation, as outlined above. Additionally, by mapping onto longer reads, extra bases were added to reads that did not necessarily belong to them.

PyroNoise also caused 13,647 changes to the reads (Table 2). The plurality of these changes were substitutions, with roughly equal numbers of insertions and deletions. Reads that did not cluster with any others – clusters of size one – did not have any changes (File S4A). To evaluate the changes, we examined the reads’ flowgrams. We found that most of the insertions and deletions (for example, File S5) resulted from changes in the flowgrams that were consistent with the known pattern of pyrosequencing errors [7]–[9].

However, when we examined substitutions, we found that most of the changes were not supported by the flowgrams (File S6A). The largest cluster formed by PyroNoise had a T → A substitution made at one position to nearly 20% of its reads, despite the fact that the corresponding flow values form two distinct groups that do not appear noisy (File S6B). Such substitutions were unlikely to be caused by pyrosequencing errors. In fact, the substitution error rate of 454 pyrosequencing is known to be far lower than the insertion or deletion rates, since substitution errors can only appear as a result of an overcall being followed by an undercall, or vice versa [7], [8].

**Figure 2:**
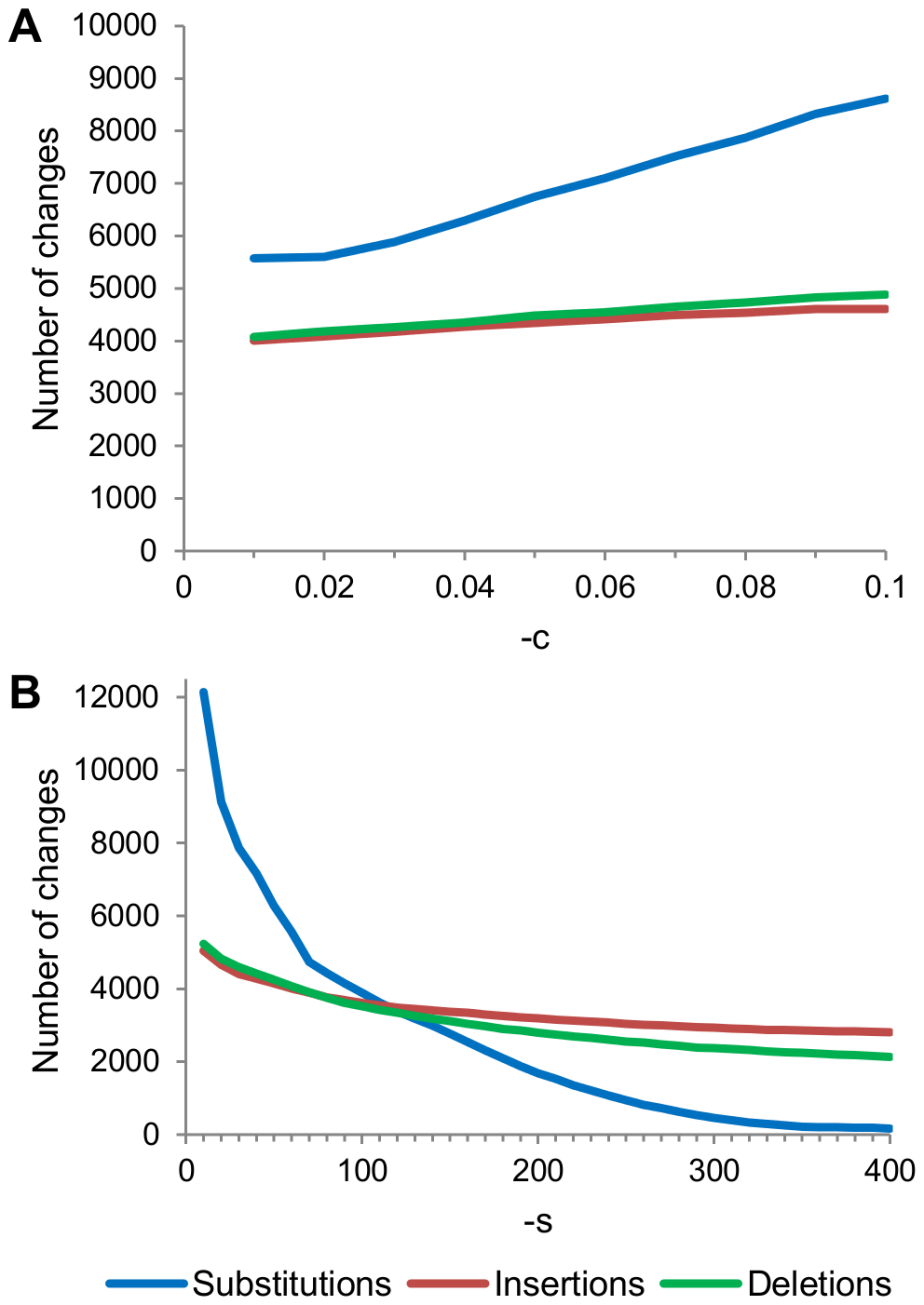
Effects of aaltering the parmeters of PyroNoise. A: The effects of altering the -c parameter in increments of 0.01, while keeping -s at the default of 60.0. B: The effects of altering -s in increments of 10, while keeping -c at the default of 0.01.

In an attempt to alter the spectrum of changes, we adjusted the values of the two parameters of PyroNoise, -c and -s. The number of substitutions was lowest when -c was 0.01, which is the minimum value as well as the default (Fig. 2A). On the other hand, increasing -s from the default of 60.0 greatly decreased the substitutions, while having less of an effect on the numbers of insertions and deletions (Fig. 2B). Therefore, keeping -c at the default value while choosing a larger value for -s caused the changes made by PyroNoise to be more consistent with the known spectrum of pyrosequencing errors.

#### Accordion Effect

**Figure 3:**
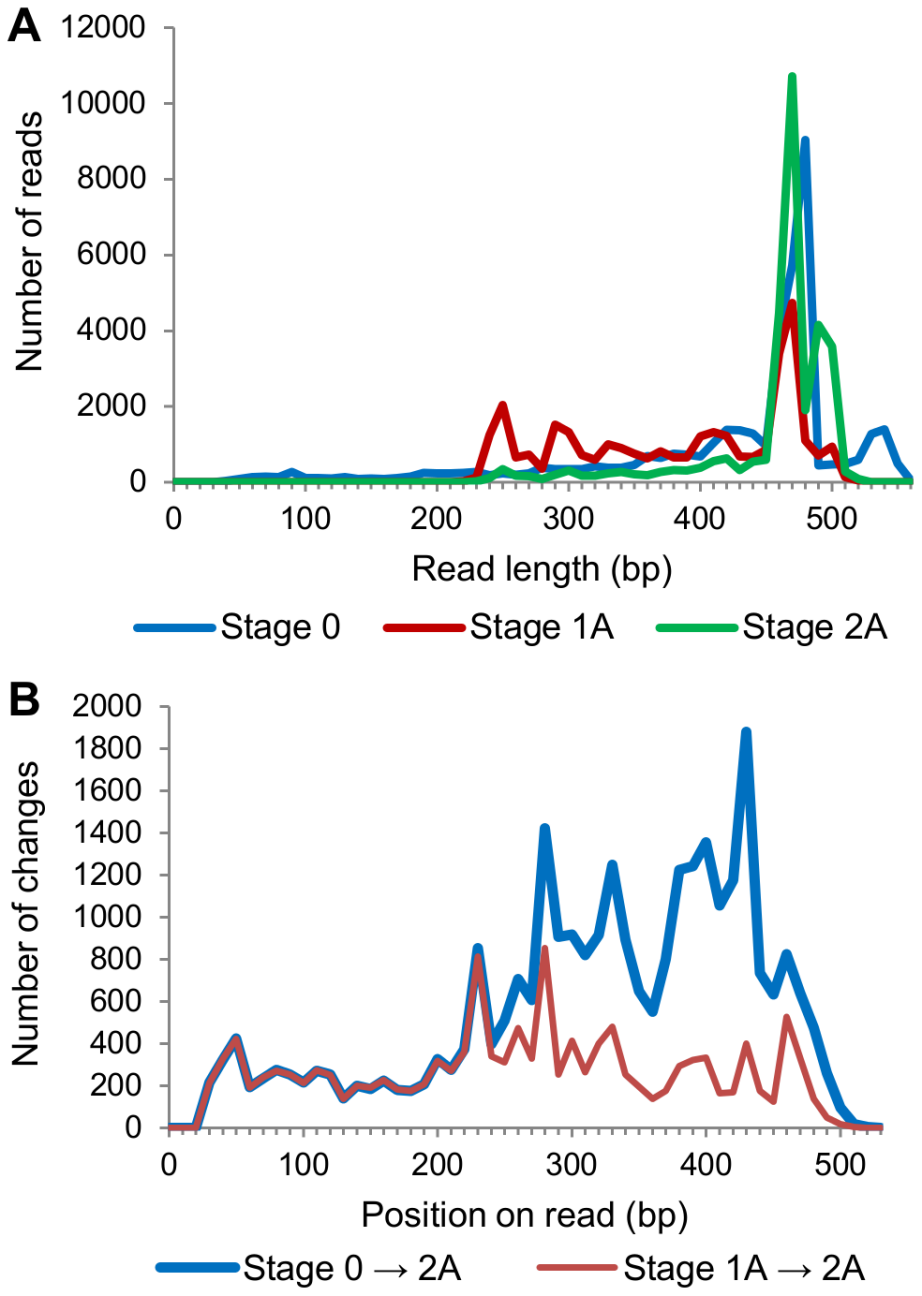
The “accordion effect” of the Filtering and PyroNoise steps. A: The original reads’ lengths (Stage 0) were reduced by the Filtering step (Stage 1A). Note the increased number of reads in the 250 – 350 bp range. After clustering by PyroNoise (Stage 2A), the read lengths were increased again, similar to Stage 0. B: The numbers of changes at each position along the Stage 2A reads, when using either Stage 0 or Stage 1A as the reference.

Next, we determined what the net effects of the first two steps of AmpliconNoise were by comparing the Stage 2A reads to the original (Stage 0) reads. As shown in Table 2, the total number of changes was nearly double the sum of the previous two steps. The source of these extra changes derived from the 3′ gap. In the Filtering step, a majority of the original reads were truncated, resulting in a decrease in read lengths (Fig. 3A). This was followed by PyroNoise, which mapped the reads onto longer ones in the clusters, thus increasing the read lengths. When comparing this analysis to that of PyroNoise alone (Stage 1A → 2A), the extra changes were seen almost exclusively at positions above 250 bp (Fig. 3B).

We call this the “accordion effect”: shrinking reads in one step, followed by lengthening them in the next step, with less than desirable results. The extra bases that were added to a read in the second step were not necessarily the same as those that were removed in the first step. In some cases, they were very different. This highlights the consequences of both the truncation criteria of the Filtering step and the mapping of reads onto longer ones in PyroNoise.

One positive effect of the Filtering and PyroNoise steps was that 89.8% of the Ns in the Stage 0 reads were changed to regular bases in Stage 2A. Most of the Ns became what appeared to be the correct bases, judging from the flow values (File S1). Although these changes were recorded as substitutions in our analysis, they should be regarded as potentially correct changes.

#### Truncation

Prior to being analyzed by SeqNoise, the clusters’ sequences need to have their mid tags (and optionally primers) removed, and to be truncated to 400 bp. The justification for this truncation is that there is an increase in error rates at the ends of reads [12]. While this is certainly true of pyrosequencing errors, those errors were just removed in the previous step. The next step is concerned with PCR single-base errors, which are not more prevalent at one end of an amplicon versus the other.

Nevertheless, for our data, we removed the mid tags and primers (one of which contained a degenerate base), and then truncated the sequences to 400 bp. This resulted in an average 3′ gap of –36.7 bp (Table 2), but no other changes to the reads. Then we combined the cluster reads from multiple samples into one of four bins (for each primer).

#### SeqNoise

The purpose of the next set of algorithms is to remove PCR single-base errors by further clustering the clusters formed by PyroNoise. SeqNoise takes the same two parameters as PyroNoise. In this case, -c has a default value of 0.08, and -s has a default value of 30.0, with values of 25.0 or 10.0 being recommended for -s when analyzing Titanium data [12].

We applied SeqNoise to our data using the default values. This resulted in 1,910 clusters, of which 839 (43.9%) consisted of a single read. The largest cluster contained 3,369 reads, derived from the largest cluster formed by PyroNoise (2,604 reads) and 331 other clusters of varying sizes from five different samples. Of the 859 clusters that were composed of multiple clusters from PyroNoise, 34.1% contained reads from more than one sample, and these clusters accounted for more than 80% of all the reads at this stage.

The average 3′ gap due to SeqNoise was minimal, but it caused 20,450 changes to the reads, most of which were substitutions (Table 2). Some of the reads were altered drastically. For example, ten reads had more than 20 substitutions each (data not shown). Clusters containing 95 reads, of which there were two, had an average of more than five changes per read (File S4B). Like with PyroNoise, the process of picking a single representative read for each cluster led to additional changes (Files S7, S8).

**Figure 4:**
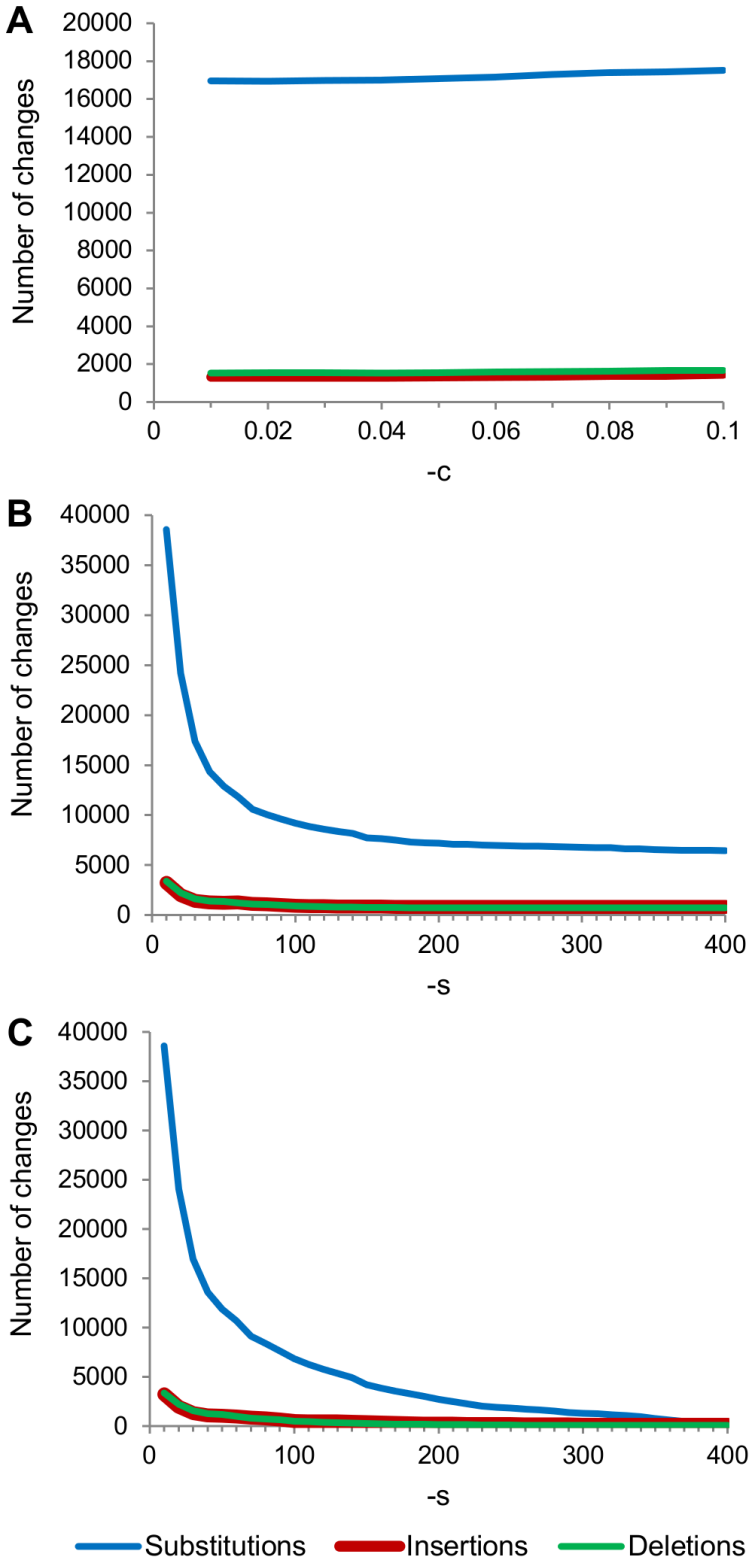
Effects of altering the parameters of SeqNoise. A: The effects of altering the -c parameter in increments of 0.01, while keeping -s at the default of 30.0. B: The effects of altering -s in increments of 10, while keeping -c at the default of 0.08. C: The effects of altering -s in increments of 10, while keeping -c at the minimum of 0.01.

We determined the effects of varying the values of the two parameters. Altering -c, while holding -s at the default of 30.0, had little apparent effect on the numbers of changes (Fig. 4A). On the other hand, increasing -s while keeping -c at the default of 0.08 caused all three types of changes to approach asymptotes, which sum to around 8,000 total changes (Fig. 4B). Using the recommended values for -s when analyzing Titanium data (25.0 and 10.0) led to a greatly increased number of changes compared to the default. Finally, with -c at the minimum value of 0.01, increasing -s caused all three types of changes to approach zero (Fig. 4C).

Unlike with PyroNoise, where we could examine the flowgrams and see if the changes were consistent with pyrosequencing errors, there was no way to look at a given change made by SeqNoise and determine whether or not it was correct. In fact, this is our principal objection to SeqNoise. One cannot examine a given DNA sequence difference and determine that it is a PCR single-base error, as opposed to being representative of actual diversity. Although SeqNoise relies on a matrix of mutation frequencies due to PCR, nature has its own matrix of mutation frequencies which follows a similar pattern: low-frequency transversions and higher-frequency transitions. Whether a given sequence difference occurred ten PCR cycles ago, as opposed to ten days ago in nature, is extremely difficult to distinguish.

#### Perseus

The final step of the AmpliconNoise pipeline is designed to remove PCR chimeras. We applied Perseus to our data using the recommended parameter values. A total of 128 clusters (6.7% of the 1,910 clusters formed by SeqNoise) were determined to be chimeras, and they consisted of 453 reads. Five of those clusters were composed of reads from multiple samples. The reads that passed Perseus, the Stage 5A reads, had no alterations compared to the Stage 4A reads (Table 2), but there were slightly fewer of them. These reads represent the final output of the AmpliconNoise pipeline.

#### Net results

Finally, we determined what the net effects of AmpliconNoise were by comparing the final denoised reads (Stage 5A) to the original reads (Stage 0). The 3′ gap was -34.3 bp on average (Table 2), ranging from -300 to +213 bp. Again, we must point out that losing hundreds of base pairs from certain reads represents a significant loss of data, and that adding hundreds of base pairs to other reads is unjustified.

The total number of changes was 42,559, which amounted to more than 1.3 per read. This total was also more than the individual steps of AmpliconNoise combined, mostly due to the accordion effect of the Filtering and PyroNoise steps. On the other hand, the number of changes was diminished by the Truncation step, which reduced the lengths of reads, and Perseus, which removed a subset of reads that had a total of 1,640 changes. It is noteworthy that the reads from the putative chimeric clusters had more than 3.6 changes per read, on average, because chimeras are not expected to be more prone to sequencing or PCR single-base errors.

#### CleanOpt

In an attempt to address some of the issues of AmpliconNoise, we wrote a filtering script, CleanOpt.pl, which produces truncated flowgrams analogous to those from CleanMinMax. Our script analyzes flowgrams flow by flow, instead of four at a time, and it truncates a flowgram immediately prior to any flow whose value is any of the following: (1) less than 0.50, with the following two flow values also less than or equal to 0.50; (2) greater than 6.49; (3) greater than or equal to 0.50 and less than 0.70. After this, the read is deleted if fewer than 360 flows remain. For our data, this resulted in the elimination of 8,903 reads (File S9A), slightly more than with CleanMinMax. The remaining flowgrams were truncated to 720 flow values.

CleanOpt also interprets sequences from the flowgrams, with minor differences from ConvertDatFasta. We determined what changes had been made to the reads after CleanOpt compared to the raw reads. Unlike after CleanMinMax, there were no substitutions or insertions to the reads (Table 2, “CleanOpt (trunc)”), because the flowgrams were truncated prior to every N and the flows that ended in “.50” were rounded down by CleanOpt. In the cases where such flow values were rounded up by the 454 software, this led to a deletion, which accounted for all of the changes at this stage. However, the fewer inconsistencies produced by this algorithm did not substantially affect the results of PyroNoise or the accordion effect (Table 2).

Another option provided by CleanOpt is to bypass the truncation criteria. The only criterion used in this case is the minimum of 360 flows, which resulted in the elimination of 3,101 reads (7.6%). Since some of the remaining reads contained Ns, the flowgrams were interpreted by CleanOpt such that an N was added to a sequence whenever there were three consecutive flows with no signal greater than 0.50.

When used with this option, CleanOpt caused minimal 3′ gaps in the reads (Table 2, “CleanOpt (no trunc)”). The large number of deletions were again due to rounding the “.50” flow values down. The substitutions and insertions were caused by flow values of 0.50 that were called by the 454 software, but were called Ns by CleanOpt. However, of the 3,035 Ns in the original reads, all but two were replaced in the reads correctly. These few differences need to be weighed against the value of the reads that were neither eliminated nor truncated in this process.

When PyroNoise was applied to these reads with the default parameters, the number of changes was substantially more (an increase of 153.8%) than it was following CleanMinMax. However, the number of substitutions was less than the numbers of insertions or deletions, which is more consistent with the known spectrum of pyrosequencing errors, and the total number of changes from Stage 0 → 2A was only slightly increased (19.2%) from that following CleanMinMax. Also, the numbers of all three types of changes from Stage 0 → 2A was less than the sum of the previous two steps. Therefore, by maintaining the reads at their original lengths with CleanOpt and bypassing the truncation criteria, no accordion effect was observed after PyroNoise.

### Qiime

The microbial ecology analysis package QIIME has its own denoising pipeline [15]. The principal algorithm, denoiser.py, was based on the original PyroNoise. It is preceded by two filtering steps (Fig. 1).

#### split_libraries

The first filtering step is to use the split_libraries.py script in QIIME to retrieve the reads. The recommended usage is to truncate the reads based on a sliding window test of quality scores, followed by the removal of any reads outside the given length parameters.

We applied split_libraries.py to our data using the recommended parameters (-w 50, -l 150, -L 550). This resulted in the extraction of 36,281 reads into four different bins (one for each primer). Another 2,220 reads were lost due to the truncation of -w occurring before the 150 bp minimum (File S9B).

We analyzed what changes had been made to these Stage 1B reads compared to the original (Stage 0) reads. The average 3′ gap was -48.1 bp (Table 2), with as many as 358 bp removed from a read. There were no other changes to the reads.

Another usage is to specify the -g option, which eliminates any read that has a window of poor quality scores. This would have resulted in the elimination of the 20,493 reads that were truncated without -g (File S9B), most of which were from our longer amplicon.

#### Pre-clustering

The next filtering step in this pipeline is to cluster the reads whose sequences are identical over the shorter read’s length. The clustering is performed by denoiser_preprocess.py. This script also retrieves the flowgrams for each of the clusters’ representative reads, which are the longest reads in each cluster, and it removes the mid tags and primers from them. It does not truncate the 3′ ends of the flowgrams based on the truncations of the previous step.

Application of denoiser_preprocess to our data without run-length encoding resulted in 22,151 clusters. As we did with AmpliconNoise, we expanded the representative reads to each of the reads in the corresponding clusters (File S2), and then determined what changes had been made to these (Stage 2B) reads. As expected, the 3′ gap was large and positive (Table 2), but there were also more than a thousand changes. This was due to the fact that, for the first time, the flowgrams were being reinterpreted into sequences at this stage. All flow values ending in “50” were rounded up, which accounted for all of the insertions and substitutions (which were conversions of Ns to regular bases). The deletions were of Ns that were due to three consecutive flows with insufficient signal, such as the example shown in File S1. Denoiser_preprocess correctly inserted 532 Ns that were due to four consecutive flow values below 0.50 (regardless of frame).

Since the truncations of the previous step were not taken into account, there was no accordion effect. Instead, the excess changes in the Stage 0 → 2B comparison were due exclusively to these differences in flowgram interpretation.

#### Denoiser

The next step is the major algorithm of this pipeline, designed to correct both pyrosequencing and PCR single-base errors. This is performed by denoiser.py, which aligns and clusters the flowgrams of the representative reads of the clusters formed in the previous step. The script takes three parameters: percent_id, low_cut-off, and high_cut-off.

We applied denoiser.py to our data using the default value of 0.97 for percent_id and specifying the --titanium option (which automatically set the low_cut-off and high_cut-off parameters to 4.00 and 5.00, respectively). A total of 1,620 clusters were formed; 66.4% of these were singletons, but they amounted to only 3.0% of the total number of reads. Of the 544 clusters containing more than one read, 62.3% were composed of reads from multiple samples, and these clusters accounted for 87.7% of all the reads.

The 544 clusters each had a single read, called the “centroid,” that represented the cluster, whose reads were listed in a separate mapping file. Another QIIME script, inflate_denoiser_output.py, was used to reconstitute the reads at this stage, in a manner analogous to our DenoiseMap script for AmpliconNoise (File S2).

Compared to the Stage 2B reads, these reads had an average 3′ gap of +30.0 bp (Table 2), ranging from -206 to +397 bp. Also, there were more than 4.3 changes per read on average, most of which were substitutions. The singletons remained unchanged, but some cluster sizes had more than 15 changes per read (File S10). A total of 556 reads each had more than 30 changes. Since the flowgram clustering by denoiser.py is done with alignment, the changes are not distinguished as to which are correcting pyrosequencing errors or PCR single-base errors. Therefore, we have no way of evaluating whether the changes made are consistent with correcting errors, other than to point to these cases and the overall large number of changes.

**Figure 5:**
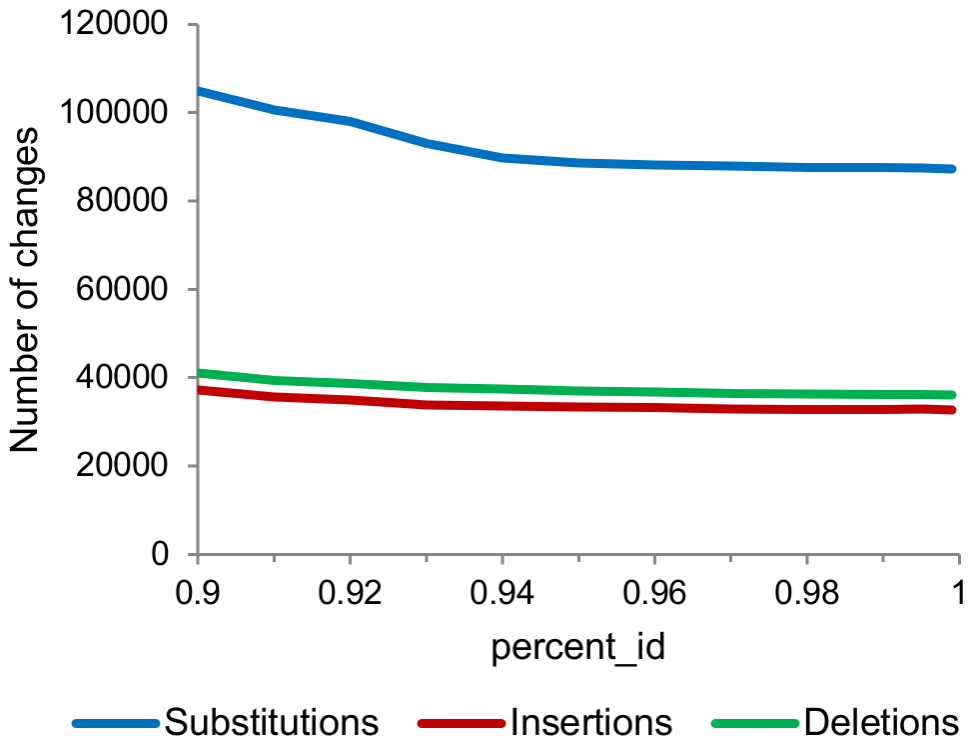
Effects of altering the percent_id parameter of denoiser.

We examined the effects of varying the parameters of denoiser.py. With two of them fixed due to the --titanium option, we could only adjust the sequence similarity clustering threshold, percent_id. Increasing this value from the default of 0.97 had little effect on the number of changes, and decreasing it also had little effect until it got below 0.94 (Fig. 5). The only way to decrease the number of changes was by not specifying the --titanium option and reducing the two cut-off parameters. At lower cut-off values, increasing the percent_id parameter further limited the changes (File S11).

We also analyzed the results of denoiser.py when applied to the reads that had been filtered using the -g option. This option had resulted in the elimination of 56.5% of the reads, but the remaining reads should have had fewer errors. However, denoiser caused a slightly increased average number of changes per read (4.6), and the changes were still mostly substitutions.

#### ChimeraSlayer

ChimeraSlayer [16] is one of the PCR chimera detection programs available in QIIME. We aligned the cluster representatives (centroids and singletons) using PyNAST [21] via the QIIME script align_seqs.py. Then we applied ChimeraSlayer, using the default parameters and the 16S Gold database [16] as the reference. It identified 41 of the clusters (2.5% of those formed by denoiser) as being chimeras, four of which contained reads from multiple samples. The chimeric clusters contained a total of 335 reads. We removed those reads from the pool to produce the Stage 4B reads, the final product of the QIIME denoising pipeline. These reads had no changes compared to the Stage 3B reads (Table 2).

#### Net results

We compared the final denoised reads from this pipeline (Stage 4B) to the original reads (Stage 0). The 3′ gap was +52.9 bp on average (Table 2), ranging from −206 to +397 bp. The total number of changes was more than 3.5 times greater than with AmpliconNoise. Part of this discrepancy was due to denoiser’s third clustering phase, which further clusters the flowgrams in a non-greedy fashion. Avoiding this phase by not specifying the --titanium option and setting the high_cut-off equal to the low_cut-off at the default value of 4.0 decreased the overall number of changes by 27.5%.

This pipeline also caused more changes than AmpliconNoise because its less stringent default filtering criteria (when avoiding the -g option) eliminated and truncated fewer reads. In addition, the reads from multiple samples were clustered together in this pipeline at the beginning. In AmpliconNoise, the reads were separated by sample prior to the PyroNoise step, and they could not be combined until after the mid tags were removed in the Truncation step. This led to fewer clusters, and thus more changes, in the QIIME denoising pipeline.

### mothur

A new denoising pipeline in mothur was recently published [17]. It includes recodings of the AmpliconNoise algorithms (Fig. 1).

#### Filtering

The filtering step in mothur offers a hybrid of the options in the two previously discussed pipelines. The algorithm trim.seqs has similar filtering criteria to that of split_libraries in QIIME, and another algorithm, trim.flows, allows for the analysis of flowgrams similar to CleanMinMax, but with more flexibility. The default of trim.flows is to require an exact match of the mid tags and primers, and to truncate flowgrams according to three criteria: (1) prior to an ambiguous base; (2) prior to a flow value between 0.50 and 0.70; and (3) to a maximum of 450 flows. After the truncations, any flowgrams with fewer than 450 flows are eliminated.

We analyzed our data with the default parameters of trim.flows. This resulted in the extraction of 25,119 reads into 56 different bins (one for each mid tag - primer combination). This meant that 38.1% of the reads were eliminated at this stage, most of which were due to a single flow value between 0.50 and 0.70 (File S9C). As we outlined in the CleanMinMax section, this criterion is unsubstantiated, but it can be adjusted (or bypassed) in this pipeline. All of the remaining flowgrams were truncated to 450 flows.

Trim.flows also interpreted sequences from the flowgrams, thus producing the Stage 1C reads. Compared to the original reads, they had a large, negative 3′ gap (Table 2), due to the strict maximum of 450 flows. There were also 399 changes to the reads, resulting from differences in interpreting the flowgrams. Trim.flows rounded all “.50” flows up, and, like CleanMinMax, it missed many Ns because it analyzed four flows at a time and in only one frame (T – A – C – G). We were able to recapitulate the output of CleanMinMax by altering the parameters of trim.flows slightly (minflows = 360, maxflows = 720, and maxhomop = 6). The resulting fasta sequences exactly matched the Stage 1A reads of AmpliconNoise.

#### shhh.flows

The next algorithm clusters the filtered flowgrams from the previous step. It is a recoding of AmpliconNoise’s PyroNoise.

We applied shhh.flows using the default parameters to the reads in each of the 56 bins separately (when we used the combined flowgram file from trim.flows, we observed several cases of mid tags being changed in reads that were sufficiently similar from different samples). This resulted in 7,304 clusters, 75.8% of which were singletons. Shhh.flows also produced files that contained the sequences for each cluster and a mapping file that listed the reads in each cluster. We used those files to reconstitute the Stage 2C reads (File S2).

Compared to the Stage 1C reads, these reads had a minimal 3′ gap (Table 2), because the reads were all approximately the same length after trim.flows. The total number of changes was far less than was seen with PyroNoise or QIIME’s denoiser (Table 2). This was due to the fact that fewer reads were analyzed, and that those reads were shorter because of the strict maximum of 450 flows in the filtering step. However, despite the lower number of changes, nearly half of them were substitutions, which is inconsistent with the known spectrum of pyrosequencing errors. Like with PyroNoise, we were able to decrease the number of substitutions by increasing the sigma parameter.

#### Accordion effect

With the reads all kept to essentially the same length after the filtering step and the minimal 3′ gap of shhh.flows, there was no accordion effect from these two steps combined (Table 2). The total number of changes was less than the sum of the two steps. In addition, 74.8% of the Ns were converted to regular bases by shhh.flows, instead of remaining deleted after trim.flows.

However, when we applied shhh.flows to the reads after the “CleanMinMax” parameters of trim.flows, the results were similar to that of PyroNoise. That is, there was a large, positive 3′ gap from Stage 1C → 2C, and this caused a substantial accordion effect. We also found that the results produced by shhh.flows, especially in this case, were inconsistent on attempted replication; with the same data, same algorithm, and same parameters, a different output was produced with each execution.

#### trim.seqs

The next step of this pipeline is to remove the mid tags and primers from the reads with trim.seqs. It is important to note that Schloss et al. [17] considered this algorithm, with a sliding window test of quality scores (similar to split_libraries in QIIME), as producing results nearly as good as the first two steps (trim.flows and shhh.flows) of this pipeline.

We used trim.seqs to remove the mid tags and primers, but performed no other trimming of the reads. The reads had no changes compared to the output from shhh.flows (Table 2).

#### shhh.seqs

This algorithm is based on SeqNoise from AmpliconNoise. With the mid tags and primers removed, the reads from multiple samples can be clustered together.

We used shhh.seqs with the default parameter, resulting in 2,554 clusters. More than half (56.6%) of these were singletons, but this amounted to just 5.6% of the total number of reads. Only 11.6% of the clusters contained reads from multiple samples, but those clusters accounted for 71.9% of all the reads at this stage. Compared to the previous stage, these reads had a minimal 3′ gap and fewer than 6,000 changes, most of which were substitutions (Table 2).

#### UCHIME

Mothur offers a variety of PCR chimera-screening programs, but Schloss et al. [17] determined that UCHIME [18] produced better results than the other algorithms. We used chimera.uchime in mothur without a reference database. It classified 145 clusters (5.7% of those formed by shhh.seqs) as being chimeras, consisting of 337 reads. Five of those clusters contained reads from multiple samples. We removed the putative chimeric reads from the pool, thus producing the Stage 5C reads, the final output of this pipeline. There were no changes from Stage 4C → 5C.

#### Net results

Compared to the original reads, the Stage 5C reads had a large, negative 3′ gap (Table 2), due the the strict maximum length of trim.flows. There were 10,257 changes to the reads, a little more than 0.4 changes per read. These numbers are far less than those of AmpliconNoise and the QIIME denoising pipeline.

**Figure 6:**
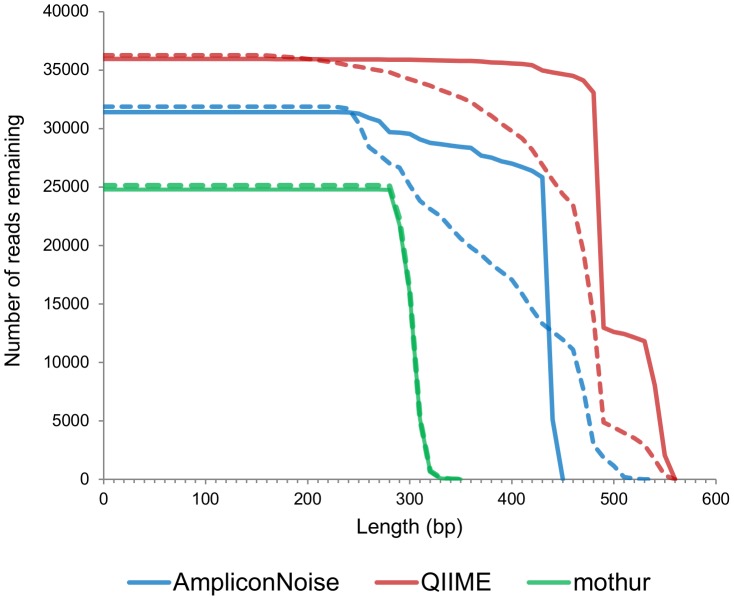
Sequence information produced by the denoising pipelines. The lengths of the reads produced at the end of the three major denoising pipelines. The total sequence information is the area under the curve. The dotted lines represent the reads (post-Filtering) that were input to each of the pipelines. Both AmpliconNoise and the QIIME denoising pipeline expand this sequence information.

The major difference in the outcome of this mothur pipeline versus the others was established in the Filtering step. By setting a minimum and maximum of 450 flows, trim.flows ensured that all the reads were approximately the same length. The reads stayed at these lengths, more or less, through the remaining steps of the pipeline, ensuring that there would be no accordion effect. Without the 3′ ends of reads, which are more prone to pyrosequencing errors, fewer changes needed to be made. However, the overall decreased number of changes was achieved at the expense of producing less sequence information than the other pipelines – 43.2% less than AmpliconNoise, and 58.7% less than the QIIME denoising pipeline (Fig. 6). Of course, the outputs of those two pipelines were slightly artificially inflated, due to the positive 3′ gaps of PyroNoise, denoiser_preprocess.py, and denoiser.py.

### Other Denoising Pipelines

Other denoising pipelines have been developed that do not rely on flowgram analysis.

#### Single-linkage preclustering

Single-linkage preclustering (SLP) [19] is designed to cluster reads containing errors with more abundant error-free reads. Since each cluster formed by SLP contains a single sequence that will be used for downstream analysis, the reads are being changed just like in the other denoising pipelines.

We began the analysis of our data by filtering with split_libraries.py in QIIME according to the criteria of Huse et al. [10]. Eliminating any reads containing an ambiguous base, with an average quality score below 25, or of anomalous length resulted in the extraction of 35,166 reads in four bins (one for each primer). Compared to the original reads, these Stage 1D reads had no changes, except that some had a small, negative 3′ gap resulting from the removal of the opposite primer at the 3′ end (Table 2).

Next, we applied the unique.seqs algorithm of mothur, which collapsed identical reads and produced an abundance file. We used the kmerdist and needledist algorithms of ESPRIT [22] with default parameters to calculate pairwise distances, as recommended [19]. Finally, we clustered the reads with slp.pl using a maximum distance of 0.02. This resulted in 1,683 clusters, 44.7% of which were singletons. Although only 15.4% of the clusters contained reads from multiple samples, they accounted for 83.3% of the reads.

Each cluster had a single representative read, which we used to reconstitute the Stage 2D reads (File S2). Compared to Stage 1D, these reads had 95,207 changes, most of which were substitutions (Table 2). Note that the numbers of insertions and deletions are given in units of events, not base pairs. We did this because the needledist calculation (based on quickdist [5]) counts insertions and deletions of any size as single differences (File S12).

**Figure 7:**
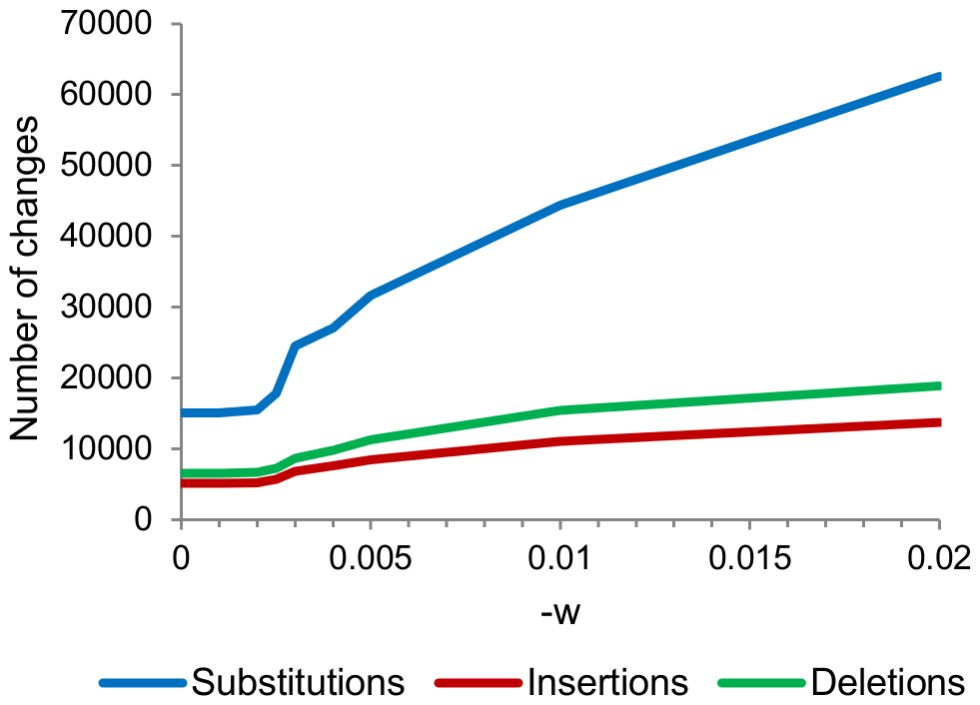
Effects of altering the -w parameter of SLP. The insertions and deletions are in units of events.

Because single-linkage allows a new read to join a cluster if it is within the given distance of any read in that cluster, there exists the possibility of increasingly divergent reads being linked together. This is known as the chaining effect. For example, with a maximum distance of 0.005, we observed chaining to a depth of six, and the reads at that depth differed from the cluster center by much more than one per 200 bp (File S13). The chaining effect also contributed to SLP’s version of the accordion effect. At a distance of zero, where only identical reads should have been clustered, 26,686 changes were made (Fig. 7). This was due to cases of divergent reads’ being linked by a shorter read that was a perfect match to both of them (File S14).

Therefore, it is important that reads analyzed by SLP be the same length to avoid the accordion effect. Huse et al. [19] accomplished this with their shorter amplicon reads by requiring that the opposite primer be present. We did not use this requirement with our data, since that would have resulted in the elimination of most of the reads from our longer amplicon. It is likely that a combination of the chaining and accordion effects caused the paradoxical increase in error rates after SLP that has been observed [12], because the analyzed reads were of different lengths.

The mothur denoising pipeline has a variation of SLP called pre.cluster, which Schloss et al. [17] considered a slightly preferable alternative to shhh.seqs in the mothur denoising pipeline. An important distinction is that pre.cluster is not a true single-linkage algorithm, because it adds reads to a cluster only if they are within the maximum distance to the cluster center. Thus, it avoids the chaining effect altogether. Pre.cluster also analyzes the aligned reads directly, thus skipping the issues arising from needledist. However, it is still not clear in a real community study that this algorithm is correcting errors as opposed to altering natural variation, nor is it clear that the changes made would improve the results of any downstream clustering process.

#### Acacia

Acacia [20] is a recently published denoising algorithm. It filters the reads based on sequences and quality scores, and then clusters them using hypothesis testing of homopolymer lengths.

We analyzed our data with Acacia using the default parameters. The 37,802 reads that passed the filtering were placed into 12,706 clusters, 80.5% of which were singletons. Although Acacia will cluster reads from multiple samples together, only 22.8% of the reads were in such clusters. All the clusters had a single consensus sequence, but each read had its own denoised sequence that sometimes varied from that of its cluster in length. We concatenated these sequences to produce the output from the Acacia pipeline.

Compared to the original reads, these reads had 20,170 changes, 38.5% of which were substitutions. Since Acacia attempts to correct only homopolymer errors, one would expect fewer substitutions. In fact, the number of substitutions was already diminished by Acacia at the expense of additional deletions (File S15). We also found that the results produced by Acacia were inconsistent on attempted replication.

### Additional validation

To ensure that these results were not specific to our particular dataset, we analyzed the dataset from a marker-based metagenomic study of three marine organisms (brown coral, orange coral, and sponge). The 16S rRNA amplicon (V6–V8) was sequenced bidirectionally (800 flows), resulting in 49,855 reads. They were processed through the denoising pipelines and produced similar results (File S16).

In the AmpliconNoise pipeline, the spectrum of changes produced by PyroNoise showed more substitutions than insertions and deletions unless the -s parameter was increased. There was a very large accordion effect, but this was avoided by using our CleanOpt script without truncations. The QIIME denoising pipeline produced more than three times as many changes as AmpliconNoise. The strict filtering criteria of mothur greatly reduced the overall number of changes, but at the expense of producing less sequence information than the first two pipelines.

## Conclusions

We have denoised a real 16S rRNA metagenomic dataset and analyzed the changes produced by each step of five denoising pipelines. Even without knowing the true sequences, we have been able to evaluate the effects of the algorithms, and we have discerned several points at which the sequence reads were changed in a manner inconsistent with removing noise. Others who utilize these pipelines should use this approach to determine how their reads are being transformed by the denoising process and to optimize the results.

Fundamentally, all five pipelines are designed to reduce the effects of noise – pyrosequencing errors, PCR single-base errors, and PCR chimeras – in metagenomic analyses. Before considering those sources of noise, we must first address the different filtering criteria that each pipeline uses.

### Filtering

Eliminating reads that are likely to contain many errors and produce erroneous OTUs in downstream analysis, as well as truncating reads prior to regions that are likely to be noisy, are good ideas. However, most of the eliminations and truncations of CleanMinMax in AmpliconNoise are due to a single unsubstantiated criterion, and it fails to truncate prior to an ambiguous base. This can be overcome using our script CleanOpt.pl, which provides the option of truncating the reads according to the intended criteria or not.

The Filtering steps in QIIME and mothur allow for additional control over this process. In mothur, trim.flows analyzes the flowgrams similar to CleanMinMax, but one has the opportunity to avoid some of the negative results by adjusting the parameters. Filtering in the QIIME pipeline is accomplished by analyzing sequences and quality scores, and the user can choose from a variety of well-established criteria. The only issue here is that the truncations of the sequences are not applied to the flowgrams, which are analyzed by denoiser. For SLP, we used QIIME’s filtering script as well, because of the control it offered.

### Pyrosequencing errors

In Roche-454 pyrosequencing, errors occur from the inaccurate determination of homopolymer lengths. The original PyroNoise algorithm addressed these errors by considering the flowgrams, from which insight could be gained about the confidence of the sequence calls. For example, suppose that two sequences differ by an insertion, with one having 2 As and the other having 3 As. If the corresponding flow values are 2.49 and 2.51, then they are likely to be the same, but the flow values could also be 1.51 and 3.49, much further apart. We consider this, the analysis of flowgrams, to be an essential part of any algorithm that is designed to denoise marker-based metagenomic data produced by Roche-454 pyrosequencing. PyroNoise (in AmpliconNoise), denoiser (in QIIME), and trim.flows (in mothur) all use the information in the flowgrams.

Any algorithm that is designed to correct pyrosequencing errors should make changes to the sequence reads that are consistent with the known spectrum of these errors – a much smaller number of base substitutions compared to insertions and deletions. We found that the PyroNoise part of AmpliconNoise caused a large number of substitutions to our data, and these changes were not supported by the flowgrams. We were able to optimize the results of this step by increasing the -s parameter, and we did the same with shhh.flows in mothur. Denoiser (in QIIME) attempts to correct both pyrosequencing errors and PCR single-base errors in a single flowgram clustering step, so we could not examine the pattern of changes at this step specifically.

Another issue arises when establishing a sequence for a cluster of reads. PyroNoise picks the longest read in each cluster as the representative. This leads to the accordion effect, in which additional changes are made to the 3′ ends of reads that were truncated in the Filtering step. If one uses our CleanOpt script without truncations prior to PyroNoise, the accordion effect is avoided. Similarly, in mothur, there is no accordion effect, because all the reads are truncated to the same length by trim.flows. Of all the denoising algorithms analyzed, only Acacia formed a true consensus for each cluster, instead of picking a representative read.

### PCR single-base errors

To identify PCR single-base errors, an algorithm must examine a given sequence difference and determine that it is an error and not representative of the actual diversity of a real community. Unfortunately, this is difficult to do. SeqNoise (in AmpliconNoise) and shhh.seqs (in mothur) use a matrix of estimated error frequencies for PCR, but the polymerases of real organisms produce a similar spectrum of mutations. In a real community study, it is unclear that these algorithms are removing noise and not forcing rare variants to join a consensus.

Another approach is to use single-linkage preclustering. The SLP algorithm was designed to remove noise from data while retaining the rare biosphere. However, single-linkage carries with it the chaining effect, in which increasingly divergent reads are clustered together. When analyzing reads of different lengths, SLP also has its own accordion effect, with dissimilar reads being linked by a shorter read, so it should only be used on reads that are the same length. A related algorithm, pre.cluster in mothur, avoids these negative effects by not allowing the chaining effect, but this algorithm would be unlikely to affect the OTU clustering process to follow.

We were able to adjust the number of changes made by SeqNoise by manipulating its two parameters. However, even if we were to calculate an expected number of PCR errors for a given reaction, it would still be inappropriate to use SeqNoise at the parameters that would produce this number of changes. We would not be able to determine if the changes made were the correct ones in a real community study. Instead, we believe that it is best to take steps to minimize the number of errors in a PCR reaction, such as by using proofreading enzymes and limiting the number of cycles. Beyond that, we recommend that no further manipulation of the data take place, and point out that PCR single-base errors are thought to contribute much less to noise than pyrosequencing errors or PCR chimeras [17].

### Truncations

The truncations of the reads, in the Filtering and later steps, have profound effects on the outcomes of the pipelines. For example, because of mothur’s truncating reads at 450 flows in the Filtering step, the reads are trimmed to the same lengths, thus avoiding any accordion effect in subsequent steps. However, this strict length requirement also caused mothur to analyze fewer reads and to produce less sequence information than the other denoising pipelines.

In a marker-based metagenomic study that includes multiple samples, the mid tags need to be removed from the 5′ ends of the reads before OTU clustering, so that one can identify the OTUs that are shared among different samples. In denoising, it is conceptually better to analyze reads from multiple samples together as much as possible, so that reads from rare taxa have a chance to cluster together. This occurs as a default in QIIME (although it can be avoided by specifying the -S option), as well as in SLP and Acacia, which explicitly offers the option of whether to cluster reads from multiple samples or not. The Filtering steps of AmpliconNoise and mothur do not remove the mid tags from the flowgrams, so the reads must be clustered separately by sample until after the Truncation step.

### Consequences of denoising

The denoising pipelines were designed to remove the effects of noise in metagenomic analyses, such as determining the number of OTUs in a sample. In fact, they were benchmarked by performing such analyses on mock communities with known components. While applying one of these denoising pipelines to a real community dataset may get us closer to the right answer, it is important to consider at what expense this is achieved. If all rare variants are removed or “corrected” to match more common sequences, then this is not a positive outcome. Nor is it a positive outcome if the taxonomic identities of certain reads are radically altered. For example, two of our reads matched slight variants of a *Stenotrophomonas* species and a *Xanthomonas* species (both order Xanthomonadales) prior to denoising, and after denoising by AmpliconNoise they were perfect matches to *Pseudomonas* (order Pseudomonadales). Another read made the opposite journey, starting as a 98% match to *Pseudomonas putida* and ending as a 100% match to *Stenotrophomonas*. Such cases illustrate the potential consequences of algorithms that do more than simply remove noise from the data.

## Materials and Methods

### Ethics statement

No specific permits were required for the described field studies. No locations were privately owned or protected in any way. No endangered or protected species were involved.

### Sample preparation and sequencing

Fourteen individual nematodes were selected from ocean sediment samples taken from off the New Hampshire coast and the Gulf of Mexico. Genomic DNA was isolated by incubating the nematodes in lysis buffer [23] at 65° for 2 hr, followed by 5 min at 95°. This resulted in the isolation of nematode DNA, along with the genomic DNA from any associated organisms.

Two regions of the bacterial 16S ribosomal RNA gene were amplified by PCR using DyNAzyme^™^ EXT DNA polymerase (Finnzymes). The PCR primers consisted of a 25-bp adaptor sequence (Roche-454), followed by a 10–11 bp multiplex identifier (mid) tag [24] that was unique for each nematode, followed by one of the following four primers: 341F - CCTACGGGAGGCAGCAG; 926R - CCGTCAATTCMTTTGAGTTT; 968F - AACGCGAAGAACCTTAC; 1401R - CGGTGTGTACAAGGCCCGGGAACG [25]. The products were purified and sequenced on the Roche-454 GS FLX Titanium platform (800 flows). Both amplicons (341F - 926R and 968F - 1401R) were sequenced bidirectionally.

The output standard flowgram files were concatenated and converted to text form using the Roche-454 Tools programs sfffile and sffinfo, respectively. The flowgram files have been submitted to the NCBI Sequence Read Archive (accession SRR653182), and the mapping file is given as File S17.

### Denoising pipelines

AmpliconNoise V1.25 was implemented according to Quince et al. [12] and the User Guide and the Tutorial that are provided with the downloaded scripts. The denoising pipeline in QIIME 1.5.0 [13] was implemented according to Reeder and Knight [15] and the QIIME website (qiime.sourceforge.net), along with the 4.29.2010 release of ChimeraSlayer [16]. The denoising pipeline in mothur v.1.25.1 [14], along with UCHIME v4.2.40 [18], was implemented according to Schloss et al. [17] and the mothur website (www.mothur.org). The SLP pipeline was implemented according to Huse et al. [19] and the MBL website (vamps.mbl.edu/resources/). Acacia was implemented according to Bragg et al. [20].

### Reconstituting reads

To evaluate the consequences of each step in the denoising process, we reconstituted the reads at each of the stages using our DenoiseMap scripts (Files S18, S19, S20, S21). The original reads (Stage 0) were given by the fasta file provided by the 454 software.

In AmpliconNoise, the Stage 1A reads were created by concatenating the fasta files created by ConvertDatFasta, which interpreted the flowgrams that were cleaned by CleanMinMax (the fasta files produced directly by CleanMinMax did not accurately reflect the reads at this stage). The other Stage 1 reads were similarly made from the fasta files produced by split_libraries (in QIIME and SLP) and trim.flows in mothur. For the remaining stages, the reads were created by matching each read from the mapping file to the corresponding cluster’s sequence in the sequence file (File S2).

When reconstituting the reads, we reattached mid tag and primer sequences to them when necessary, beginning with Stage 3A in AmpliconNoise, Stage 1B in QIIME, Stage 3C in mothur, Stage 1D in SLP, and Stage 1E in Acacia.

### Quantifying changes

To determine what changes had been made to the reads, we used our script AlignClusMus.pl (File S22). In brief, this script takes two multifasta files, a query and a reference, as inputs. It aligns each sequence in the query file to the corresponding sequence from the reference file using ClustalW 2.1 [26], with a reduced gap-opening penalty (-gapopen = 1). The 3′ gap is recorded, and the extra bases are removed from the longer sequence. Then, another alignment is performed using MUSCLE v3.8.31 [27], again with a reduced gap-opening parameter (-gapopen -100). This alignment result is analyzed for substitutions, insertions, and deletions. The insertions and deletions are recorded in units of both base-pairs and events. In this paper, these are given in units of base-pairs unless otherwise indicated.

For our data, allowance was made for the ambiguous base in the 926R primer. A sample output from AlignClusMus.pl is given as File S23.

### CleanOpt

Our filtering script, CleanOpt.pl (File S24), analyzes the individual flows of each flowgram retrieved by SplitKeys, up to the last flow called by the 454 software. It provides the option of truncating the flowgrams according to the intended criteria of AmpliconNoise or not. In either case, the format of the resulting files matches that of CleanMinMax, so that any files can be further analyzed by the PyroNoise step of AmpliconNoise.

## Supporting Information

File S1 **Flowgram interpretation by AmpliconNoise.** A small selection of eight flow values from the flowgram of a read, along with the interpreted sequence at three stages. The first flow, of the nucleotide T, caused a light emission of 1.91 units in this particular well of the sequencing plate. The 454 software (Stage 0) interpreted this signal as corresponding to the sequencing strand incorporating two Ts. Next, the nucleotide A was flowed, but the low signal (0.01) meant that A was not the next nucleotide on the sequencing strand. Similarly, the 454 software called two Cs for the next flow value of 1.66. After this, none of the next three flows had a sufficient signal to call a base. Since, following the two Cs, the next base must have been one of G, T, or A, the 454 software called an N. Normal base calling followed this with the last two flows shown. The AmpliconNoise script CleanMinMax.pl, because it analyzed only one frame of four flows (T - A - C - G), did not notice the three flow values with insufficient signal. Therefore, it did not truncate the flowgram prior to these flows, and ConvertDatFasta.pl interpreted this section of the flowgram such that the N was deleted (Stage 1A). After flowgram clustering by PyroNoise (Stage 2A), the putatively correct base was inserted into the sequence.
(PDF)

File S2 **The files used to reconstitute the reads at each stage of the denoising pipelines.**
(PDF)

File S3 **Alignment of a cluster of three reads formed by PyroNoise.** A: The longest read was chosen as the representative for the cluster, even though this caused a deletion in both of the other reads. B: The flow values suggest that the correct homopolymer length was more likely to be three than four.
(PDF)

File S4 **Changes caused by PyroNoise and SeqNoise.** A: The total number of changes – substitutions, insertions, and deletions – was determined by comparing each read post-PyroNoise (Stage 2A) to that pre-PyroNoise (Stage 1A). These changes were summed and divided by the total number of reads in each cluster size. Inset: A close-up of the smaller cluster sizes. B: The same analysis performed on the reads post-SeqNoise (Stage 4A) compared to those pre-SeqNoise (Stage 3A).
(PDF)

File S5 **A deletion made by PyroNoise.** A small section of the flowgrams of a read (454 accession number “FY1WZ”) that had a deletion, along with two other reads with which it was clustered. The flow value of 4.80 was judged by PyroNoise as not being distinct from the corresponding flow values of the other reads with which it was clustered. Therefore, this value was changed to 4, resulting in a reduction of the homopolymer of five Ts to four.
(PDF)

File S6 **A substitution made by PyroNoise.** A: A small section of the flowgrams of a read (“A81VH”) that had a T → A substitution. In this case, two flow values were changed: the T from 1.94 to 1, and the A from 0.95 to 2. B: A more systematic view of the cluster shown in A. Flow values 421 (T) and 422 (A) were recorded for the reads in that cluster. Although neither of the two groups appears noisy, PyroNoise determined that the group at (2T, 1A) had pyrosequencing errors and changed those reads to match the others at (1T, 2A).
(PDF)

File S7 **Alignment of a cluster of three reads formed by SeqNoise.** Each of the three reads was a singleton after PyroNoise. Because of the choice of “HJ69D” as the representative read, a T → C substitution was made to both of the other reads.
(PDF)

File S8 **Alignment of a cluster of three reads formed by SeqNoise.** The three reads differ in three positions, at each of which two of the reads agree. None of the three reads matches the dominant base at all three positions. Therefore, forming a true consensus requires a sequence that matches none of the three.
(PDF)

File S9 **Results of other pipelines’ Filtering steps.** A: CleanOpt. B: split_libraries (QIIME). C: trim.flows (mothur).
(PDF)

File S10 **Changes caused by denoiser.** A: The total number of changes per read was calculated for each cluster size. Inset: A close-up of the smaller cluster sizes.
(PDF)

File S11 **Effects of altering the cut-off parameters of denoiser.** By setting low_cut-off and high_cut-off equal to each other, the third clustering phase of denoiser is avoided. A: percent_id  =  0.97. B: percent_id  =  0.99.
(PDF)

File S12 **Alignment of two reads in SLP.** The needledist algorithm of Esprit, based on quickdist, considers insertions and deletions of any size as being a single event. Therefore, it calculates the distance between these two reads as 1 mismatch / (212 matches + 1 mismatch)  =  1/213  =  0.004695. These reads will be clustered by SLP if the cluster width parameter, -w, is greater than this distance.
(PDF)

File S13 **Pairwise alignment of three reads clustered by SLP.** A: A chain of reads that are clustered together by SLP. B: The distance between the first and last reads far exceeds the cluster width of 0.005, because of the chaining effect.
(PDF)

File S14 **Pairwise alignments of three reads clustered by SLP.** A, B: Pairs of reads that are identical over the shorter read’s length. When using a width of 0, the first and third reads are clustered together via the second read, despite their nonzero distance (C).
(PDF)

File S15 **Alignment of two reads clustered by Acacia.** At two positions where the reads disagree, Acacia caused a deletion in both, instead of creating a substitution in one of the them. The reason for the deletion of the C near the 3′ end of the read “FH6HB” is unknown.
(PDF)

File S16 **Changes made at each step of the denoising pipelines to an independent dataset.**
(PDF)

File S17 **The mapping file for our dataset.** The primer sequences are given, followed by the corresponding mid tag sequences for each of the fourteen samples.
(CSV)

File S18 **DenoiseMapAmpNoise.pl script.**
(TXT)

File S19 **DenoiseMapQiime.pl script.**
(TXT)

File S20 **DenoiseMapMothur.pl script.**
(TXT)

File S21 **DenoiseMapSLP.pl script.**
(TXT)

File S22 **AlignClusMus.pl script.**
(TXT)

File S23 **A sample output from AlignClusMus.pl.**
(XLS)

File S24 **CleanOpt.pl script.**
(TXT)

File S25 **Rank-abundance curves.** A: The output reads from each of the denoising pipelines, as well as the original reads, were clustered (separately) to form 97% OTUs by the QIIME script pick_otus.py. B: A close-up of the 15 most abundant OTUs. Note the non-logarithmic y-axis.
(PDF)

File S26 **Rank-abundance curves of the multi-stage pipelines.** A: AmpliconNoise. B: QIIME. C: mothur. D: SLP.
(PDF)
