# Supplementary material for: Assessing the Consequences of Denoising Marker-Based Metagenomic Data
Source: PLoS One. 2013 Mar 25;8(3):e60458. doi: 10.1371/journal.pone.0060458 (PMC3607570; doi:10.1371/journal.pone.0060458)
Supplement: File S1 — Flowgram interpretation by AmpliconNoise. A small selection of eight flow values from the flowgram of a read, along with the interpreted sequence at three stages. The first flow, of the nucleotide T, caused a light emission of 1.91 units in this particular well of the sequencing plate. The 454 software (Stage 0) interpreted this signal as corresponding to the sequencing strand incorporating two Ts. Next, the nucleotide A was flowed, but the low signal (0.01) meant that A was not the next nucleotide on the sequencing strand. Similarly, the 454 software called two Cs for the next flow value of 1.66. After this, none of the next three flows had a sufficient signal to call a base. Since, following the two Cs, the next base must have been one of G, T, or A, the 454 software called an N. Normal base calling followed this with the last two flows shown. The AmpliconNoise script CleanMinMax.pl, because it analyzed only one frame of four flows (T - A - C - G), did not notice the three flow values with insufficient signal. Therefore, it did not truncate the flowgram prior to these flows, and ConvertDatFasta.pl interpreted this section of the flowgram such that the N was deleted (Stage 1A). After flowgram clustering by PyroNoise (Stage 2A), the putatively correct base was inserted into the sequence. (PDF) [file pone.0060458.s001.pdf]

Flow:	T	A	C	G	T	A	C	G
Flow values:	1.91	0.01	1.66	0.19	0.46	0.06	1.90	1.93
>Stage 0	TT		CC		N		CC	GG
>Stage 1A	TT		CC				CC	GG
>Stage 2A	TT		CC		T		CC	GG
