# Supplementary material for: Assessing the Consequences of Denoising Marker-Based Metagenomic Data
Source: PLoS One. 2013 Mar 25;8(3):e60458. doi: 10.1371/journal.pone.0060458 (PMC3607570; doi:10.1371/journal.pone.0060458)
Supplement: File S2 — The files used to reconstitute the reads at each stage of the denoising pipelines. (PDF) [file pone.0060458.s002.pdf]

| Stage | Sequence file | Mapping file |
|---|---|---|
| AmpliconNoise | | |
| 1A | <sample>.fa | N/A |
| 2A | <sample>_s60_c01_cd.fa | <sample>_s60_c01.mapping |
| 3A | <sample>_s60_c01_cd_tr400.fa | <sample>_s60_c01.mapping |
| 4A | all_s60_c01_cd_tr400_s30_c08_cd.fa | all_s60_c01_cd_tr400_s30_c08_cd.mapping |
| 5A | all_s60_c01_cd_tr400_s30_c08_Good.fa | all_s60_c01_cd_tr400_s30_c08_cd.mapping |
| QIIME | | |
| 1B | seqs.fna | N/A |
| 2B | prefix_dereplicated.fasta | prefix_mapping.txt |
| 3B[1], 4B[1,2] | singletons.fasta, centroids.fasta | denoiser_mapping.txt |
| mothur | | |
| 1C | <bin>.flow.fasta | N/A |
| 2C | <bin>.flow.shhh.fasta | <bin>.flow.shhh.names |
| 3C | <bin>.flow.shhh.trim.fasta | <bin>.flow.shhh.trim.names |
| 4C, 5C[3] | <bin>.flow.shhh.trim.shhh.fasta | <bin>.flow.shhh.trim.shhh.names |
| SLP | | |
| 1D | seqs.fna | N/A |
| 2D | seqs.unique.slp.unique.fa | seqs.unique.slp.names |
| Acacia | | |
| 1E | <bin>_all_tags.seqOut | N/A |

[1]Reconstituting performed by inflate_denoiser_output.py.
[2]Chimeric reads excluded based on output from ChimeraSlayer.
[3]Chimeric reads excluded based on output from UCHIME.
