# Supplementary material for: Assessing the Consequences of Denoising Marker-Based Metagenomic Data
Source: PLoS One. 2013 Mar 25;8(3):e60458. doi: 10.1371/journal.pone.0060458 (PMC3607570; doi:10.1371/journal.pone.0060458)
Supplement: File S3 — Alignment of a cluster of three reads formed by PyroNoise. A: The longest read was chosen as the representative for the cluster, even though this caused a deletion in both of the other reads. B: The flow values suggest that the correct homopolymer length was more likely to be three than four. (PDF) [file pone.0060458.s003.pdf]

A

```
cluster             AGACTCGACGTCCTACGGGAGGCAGCAGTGAGGAATATTGGTCAATGGGTGCAAGCCTGA
GZIPSVE01DKJ6F      AGACTCGACGTCCTACGGGAGGCAGCAGTGAGGAATATTGGTCAATGGGTGCAAGCCTGA
GZIPSVE02G2SK0      AGACTCGACGTCCTACGGGAGGCAGCAGTGAGGAATATTGGTCAATGGGTGCAAGCCTGA
GZIPSVE02F8LPD      AGACTCGACGTCCTACGGGAGGCAGCAGTGAGGAATATTGGTCAATGGGTGCAAGCCTGA
                    ************************************************************

cluster             ACCAGCCATCCCGCGTGAAGGACGACTGCCCTATGGGTTGTAAACTTCTTTTGTATAGGG
GZIPSVE01DKJ6F      ACCAGCCATCCCGCGTGAAGGACGACTGCCCTATGGGTTGTAAACTTCTTTTGTATAGGG
GZIPSVE02G2SK0      ACCAGCCATCCCGCGTGAAGGACGACTGCCCTATGGGTTGTAAACTTCTTTTGTATAGGG
GZIPSVE02F8LPD      ACCAGCCATCCCGCGTGAAGGACGACTGCCCTATGGGTTGTAAACTTCTTTTGTATAGGG
                    ************************************************************

cluster             ATAAACCTACCCTCGTGAGGGTAGCTGAAGGTACTATACGAATAAGCACCGGCTAACTCC
GZIPSVE01DKJ6F      ATAAACCTACCCTCGTGAGGGTAGCTGAAGGTACTATACGAATAAGCACCGGCTAACTCC
GZIPSVE02G2SK0      ATAAACCTACCCTCGTGAGGGTAGCTGAAGGTACTATACGAATAAGCACCGGCTAACTCC
GZIPSVE02F8LPD      ATAAACCTACCCTCGTGAGGGTAGCTGAAGGTACTATACGAATAAGCACCGGCTAACTCC
                    ************************************************************

cluster             GTGCCAGCAGCCGCGGTAATACGGAGGGTGCAAGCGTTATCCGGATTTTATTGGGTTTAA
GZIPSVE01DKJ6F      GTGCCAGCAGCCGCGGTAATACGGAGGGTGCAAGCGTTATCCGGATTTTATTGGGTTTAA
GZIPSVE02G2SK0      GTGCCAGCAGCCGCGGTAATACGGAGGGTGCAAGCGTTATCCGGATTT-ATTGGGTTTAA
GZIPSVE02F8LPD      GTGCCAGCAGCCGCGGTAATACGGAGGGTGCAAGCGTTATCCGGATTT-ATTGGGTTTAA
                    ************************************************ ***********

cluster             AGGGTCCGTAGGCGGACTTATAAGTCAGTGGTGAAAGCCTGTCGCTTAACGATAGAACTG
GZIPSVE01DKJ6F      AGGGTCCGTAGGCGGACTTATAAGTCAGTGGTGAAAGCCTGTCGCTTAACGATAGAACTG
GZIPSVE02G2SK0      AGGGTCCGTAGGCGGACTTATAAGTCAGTGGTGAAAGCCTGTCGCTTAACGATAGAACTG
GZIPSVE02F8LPD      AGGGTCCGTAGGCGGACTTATAAGTCAGTGGTGAAAGCCTGTCGCTTAACGATAGAACTG
                    ************************************************************

cluster             CCATTGATACTGTAAGTCTTGAGTATATTTGAGGTAGCTGGAATAAGTAGTGTAGCGGTG
GZIPSVE01DKJ6F      CCATTGATACTGTAAGTCTTGAGTATATTTGAGGTAGCTGGAATAAGTAGTGTAGCGGTG
GZIPSVE02G2SK0      CCATTGATACTGTAAGTCTTGAGTATATTTGAGGTAGCTGGAATAAGTAGTGTAGCGGTG
GZIPSVE02F8LPD      CCATTGATACTGTAAGTCTTGAGTATATTTGAGGTAGCTGGAATAAGTAGTGTAGCGGTG
                    ************************************************************

cluster             AAATGCATAGATATTACTTAGAACACCAATTGCGAAGGCAGGTTACCAAGATATAACTGA
GZIPSVE01DKJ6F      AAATGCATAGATATTACTTAGAACACCAATTGCGAAGGCAGGTTACCAAGATATAACTGA
GZIPSVE02G2SK0      AAATGCA-----------------------------------------------------
GZIPSVE02F8LPD      AAATGCATAGATATTACTTAGAACACCAATTGCGAAGGCAGGTTACCAAGATATAACTGA
                    *******

cluster             CGCTGAGGGACGAAAGCG
GZIPSVE01DKJ6F      CGCTGAGGGACGAAAGCG
GZIPSVE02G2SK0      ------------------
GZIPSVE02F8LPD      CGCTGAGGGACGAAAG--
```

B

```
              G       T       A       C       G       T       A       C       G       T

>DKJ6F                2.08    0.19    0.94    0.00    0.07    3.60    1.25    0.14    0.26    1.78
              GG              A                       TTTT    A                       TT

>G2SK0                2.30    0.16    1.26    0.06    0.15    3.28    0.91    0.08    0.31    1.84
              GG              A                       TTT     A                       TT

>F8LPD                2.04    0.16    0.89    0.06    0.08    3.29    1.01    0.18    0.21    1.87
              GG              A                       TTT     A                       TT
```
