# Supplementary figures and images for: Assessing the Consequences of Denoising Marker-Based Metagenomic Data
Source: PLoS One. 2013 Mar 25;8(3):e60458. doi: 10.1371/journal.pone.0060458 (PMC3607570; doi:10.1371/journal.pone.0060458)

**A**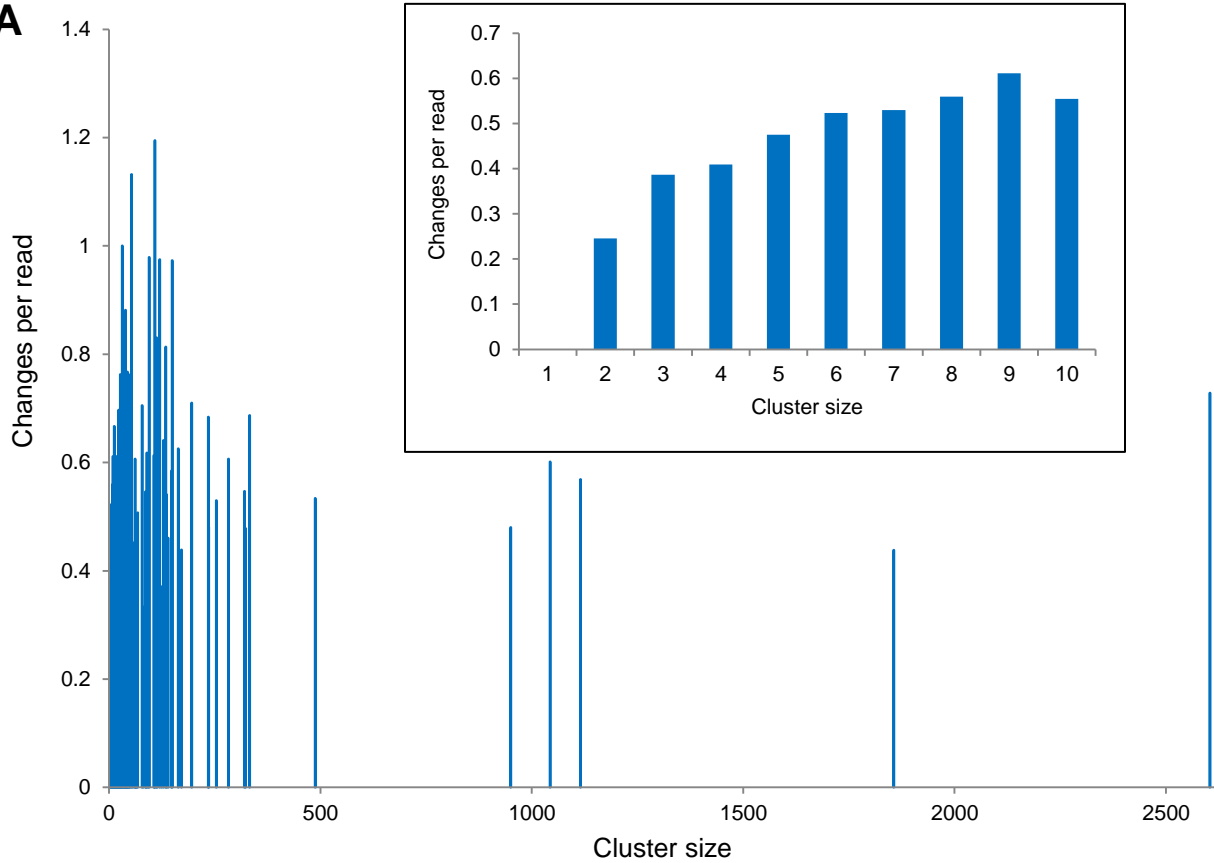**B**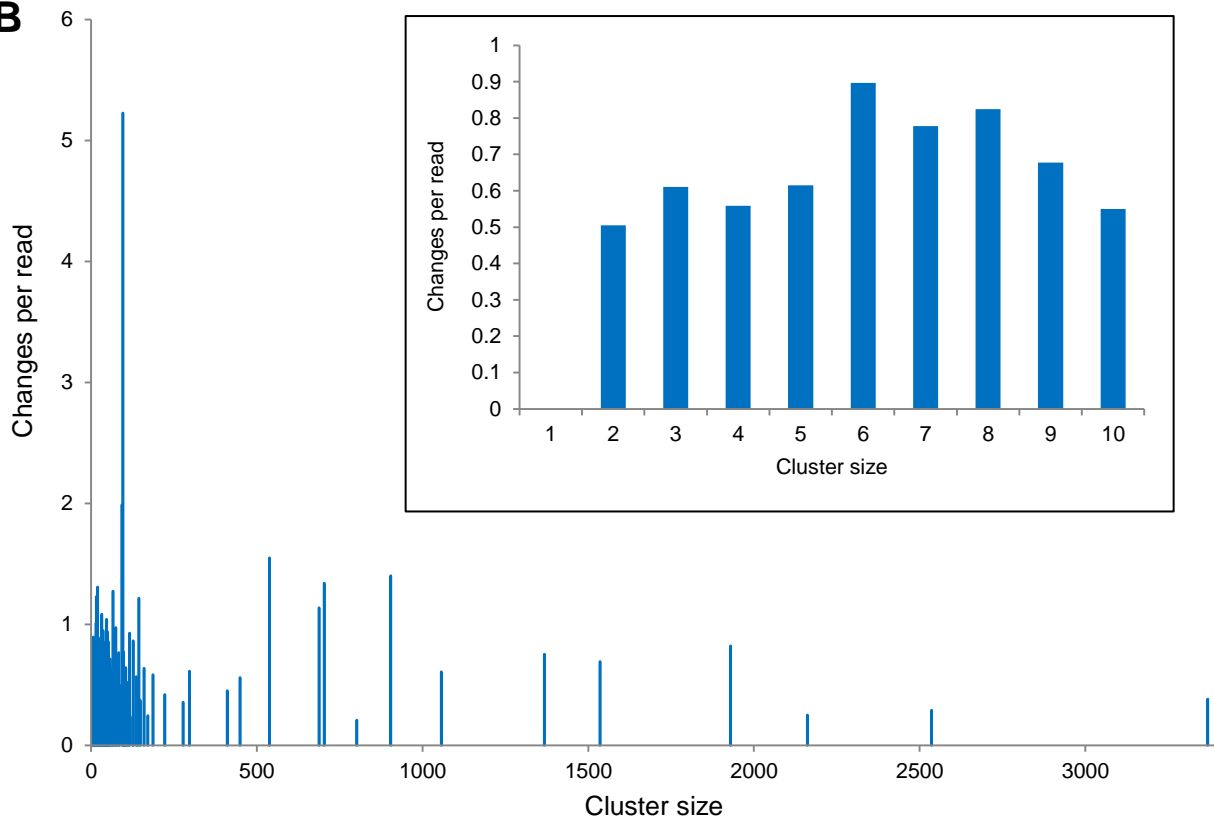

Supplement: File S4 — Changes caused by PyroNoise and SeqNoise. A: The total number of changes – substitutions, insertions, and deletions – was determined by comparing each read post-PyroNoise (Stage 2A) to that pre-PyroNoise (Stage 1A). These changes were summed and divided by the total number of reads in each cluster size. Inset: A close-up of the smaller cluster sizes. B: The same analysis performed on the reads post-SeqNoise (Stage 4A) compared to those pre-SeqNoise (Stage 3A). (PDF) [file pone.0060458.s004.pdf]

**A**

|          |      |      |      |      |      |      |
|----------|------|------|------|------|------|------|
| Flow:    | C    | G    | T    | A    | C    | G    |
| >A81VH   | 0.12 | 0.14 | 1.94 | 0.95 | 1.21 | 1.00 |
|          |      |      | TT   | A    | C    | G    |
| >BDVHW   | 0.19 | 0.12 | 1.10 | 2.07 | 1.03 | 0.95 |
|          |      |      | T    | AA   | C    | G    |
| >DGID9   | 0.14 | 0.14 | 1.04 | 2.05 | 1.18 | 0.97 |
|          |      |      | T    | AA   | C    | G    |
| >cluster |      |      | T    | AA   | C    | G    |

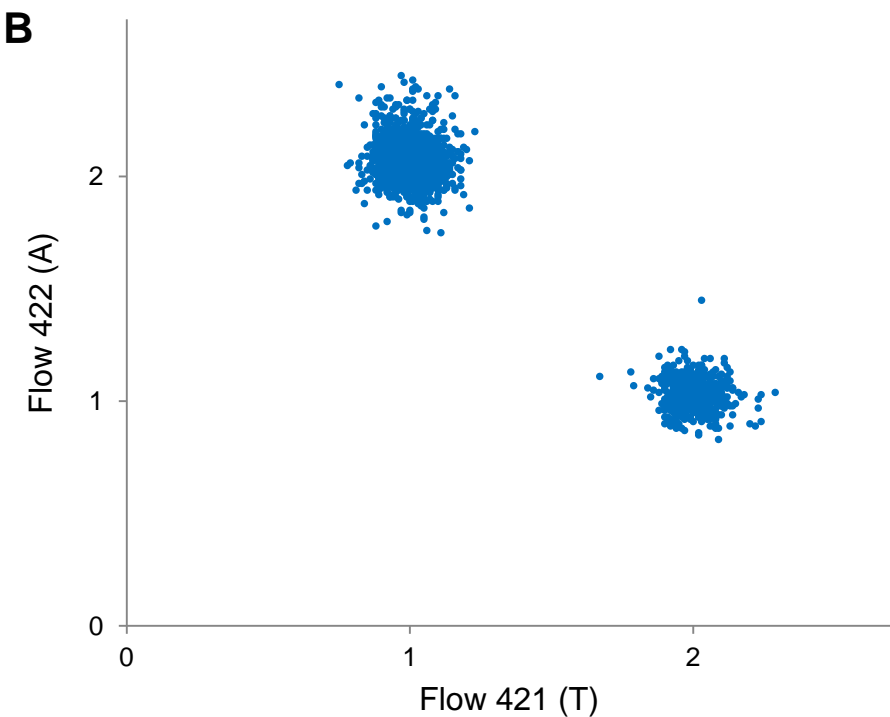

Supplement: File S6 — A substitution made by PyroNoise. A: A small section of the flowgrams of a read (“A81VH”) that had a T → A substitution. In this case, two flow values were changed: the T from 1.94 to 1, and the A from 0.95 to 2. B: A more systematic view of the cluster shown in A. Flow values 421 (T) and 422 (A) were recorded for the reads in that cluster. Although neither of the two groups appears noisy, PyroNoise determined that the group at (2T, 1A) had pyrosequencing errors and changed those reads to match the others at (1T, 2A). (PDF) [file pone.0060458.s006.pdf]

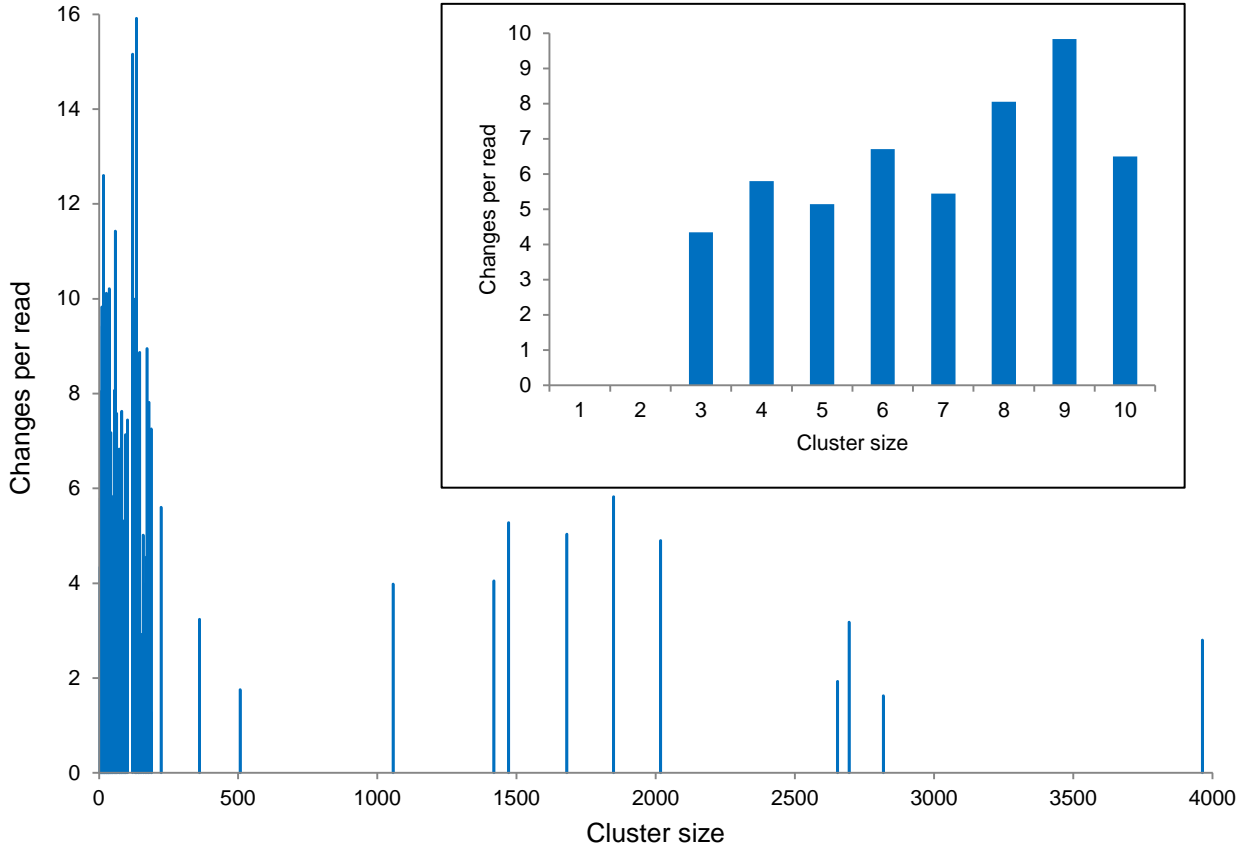

Supplement: File S10 — Changes caused by denoiser. A: The total number of changes per read was calculated for each cluster size. Inset: A close-up of the smaller cluster sizes. (PDF) [file pone.0060458.s010.pdf]

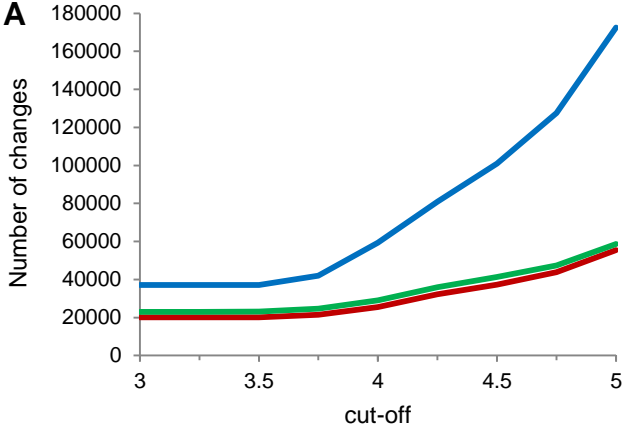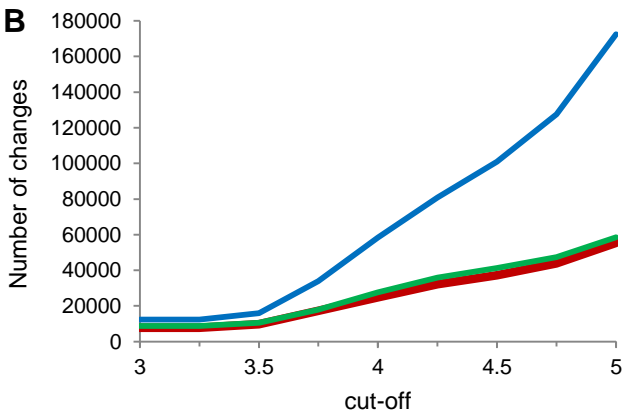

— Substitutions — Insertions — Deletions

Supplement: File S11 — Effects of altering the cut-off parameters of denoiser. By setting low_cut-off and high_cut-off equal to each other, the third clustering phase of denoiser is avoided. A: percent_id = 0.97. B: percent_id = 0.99. (PDF) [file pone.0060458.s011.pdf]

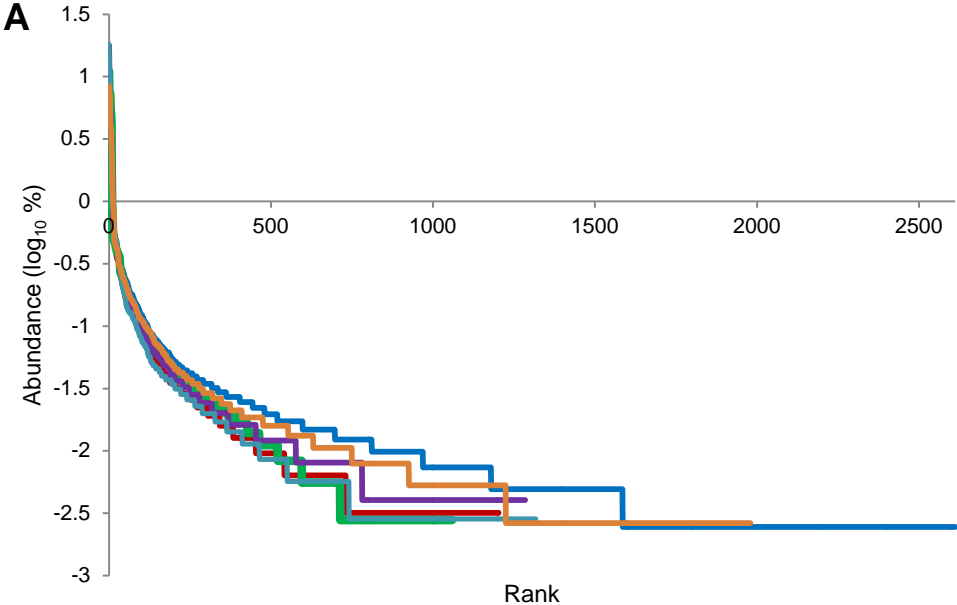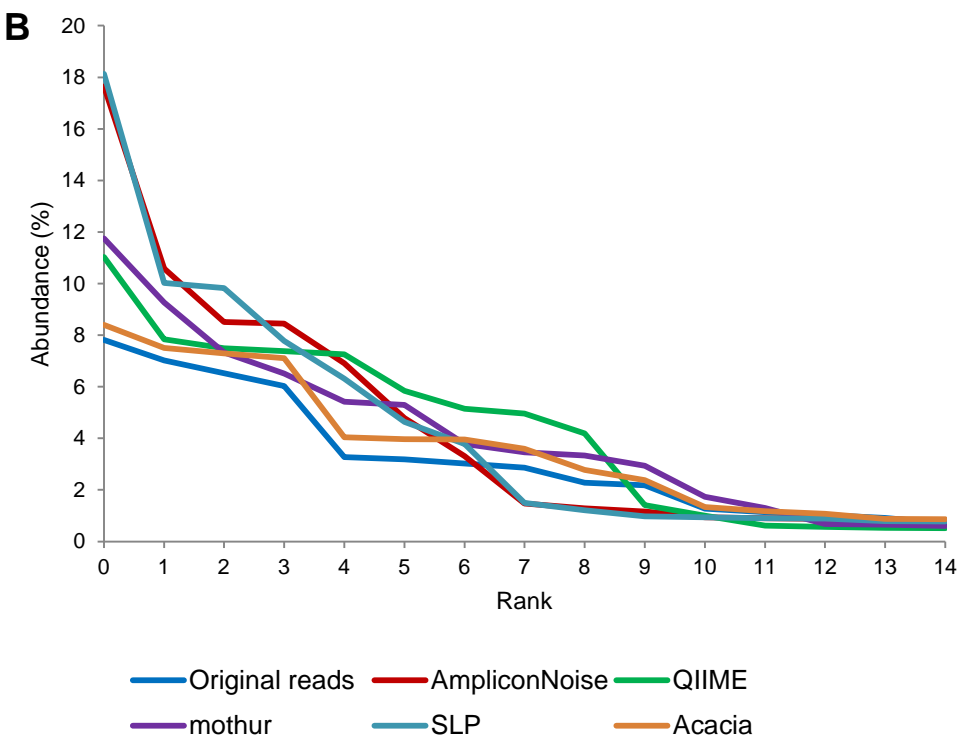

Supplement: File S25 — Rank-abundance curves. A: The output reads from each of the denoising pipelines, as well as the original reads, were clustered (separately) to form 97% OTUs by the QIIME script pick_otus.py. B: A close-up of the 15 most abundant OTUs. Note the non-logarithmic y-axis. (PDF) [file pone.0060458.s025.pdf]

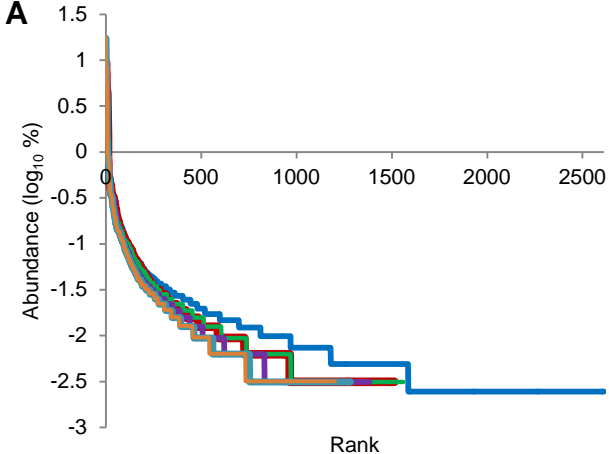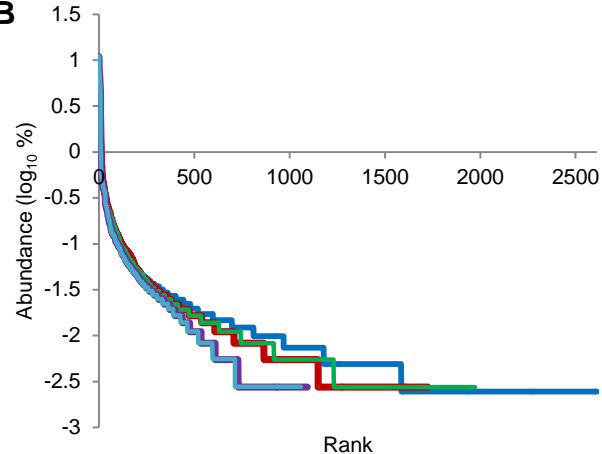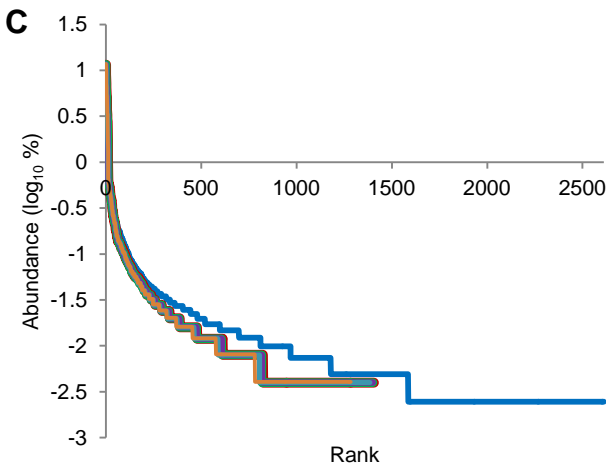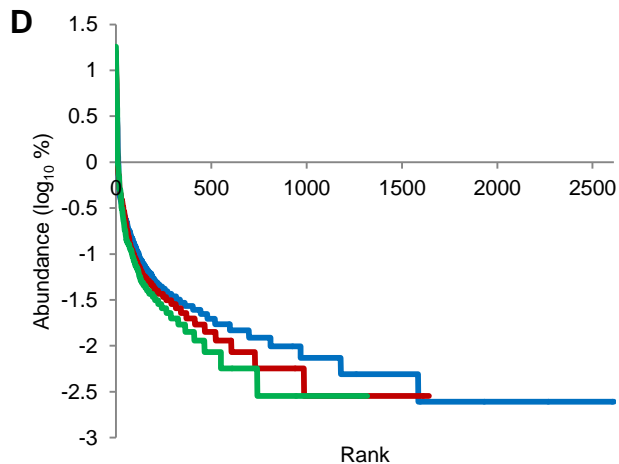

Supplement: File S26 — Rank-abundance curves of the multi-stage pipelines. A: AmpliconNoise. B: QIIME. C: mothur. D: SLP. (PDF) [file pone.0060458.s026.pdf]
