# Supplementary material for: Assessing the Consequences of Denoising Marker-Based Metagenomic Data
Source: PLoS One. 2013 Mar 25;8(3):e60458. doi: 10.1371/journal.pone.0060458 (PMC3607570; doi:10.1371/journal.pone.0060458)
Supplement: File S5 — A deletion made by PyroNoise. A small section of the flowgrams of a read (454 accession number “FY1WZ”) that had a deletion, along with two other reads with which it was clustered. The flow value of 4.80 was judged by PyroNoise as not being distinct from the corresponding flow values of the other reads with which it was clustered. Therefore, this value was changed to 4, resulting in a reduction of the homopolymer of five Ts to four. (PDF) [file pone.0060458.s005.pdf]

Flow:	C	G	T	A	C	G
>FY1WZ	1.03	1.94	4.80	0.17	0.01	0.70
	C	GG	TTTTT			G
>J3MF7	1.16	1.95	4.34	0.21	0.09	0.85
	C	GG	TTTT			G
>IYP1C	0.95	1.86	4.10	0.19	0.10	0.88
	C	GG	TTTT			G
>cluster	C	GG	TTTT			G
