# Supplementary material for: Assessing the Consequences of Denoising Marker-Based Metagenomic Data
Source: PLoS One. 2013 Mar 25;8(3):e60458. doi: 10.1371/journal.pone.0060458 (PMC3607570; doi:10.1371/journal.pone.0060458)
Supplement: File S7 — Alignment of a cluster of three reads formed by SeqNoise. Each of the three reads was a singleton after PyroNoise. Because of the choice of “HJ69D” as the representative read, a T → C substitution was made to both of the other reads. (PDF) [file pone.0060458.s007.pdf]

```
cluster            AGCACTGTAGCCGTCAATTCATTTGAGTTTTAATCTTGCGACCGTACTCCCCAGGCGGTC
GZIPSVE02HJ69D     AGCACTGTAGCCGTCAATTCATTTGAGTTTTAATCTTGCGACCGTACTCCCCAGGCGGTC
GZIPSVE01C6P61     AGCACTGTAGCCGTCAATTCATTTGAGTTTTAATCTTGCGACCGTACTCCCCAGGCGGTC
GZIPSVE01CRUN5     AGCACTGTAGCCGTCAATTCATTTGAGTTTTAATCTTGCGACCGTACTCCCCAGGCGGTC
                   ************************************************************

cluster            AACTTAATGCGTTAGCTGCGACACAGAGGGGATCAACACCCCCTGCACCTAGTTGACATC
GZIPSVE02HJ69D     AACTTAATGCGTTAGCTGCGACACAGAGGGGATCAACACCCCCTGCACCTAGTTGACATC
GZIPSVE01C6P61     AACTTAATGCGTTAGCTGCGACACAGAGGGGATCAACACCCCCTGCACCTAGTTGACATC
GZIPSVE01CRUN5     AACTTAATGCGTTAGCTGCGACACAGAGGGGATCAACACCCCCTGCACCTAGTTGACATC
                   ************************************************************

cluster            GTTTACGGCGTGGACTACCAGGGTATCTAATCCTGTTTGCTCCCCACGCTTTCGCGCCTC
GZIPSVE02HJ69D     GTTTACGGCGTGGACTACCAGGGTATCTAATCCTGTTTGCTCCCCACGCTTTCGCGCCTC
GZIPSVE01C6P61     GTTTACGGCGTGGACTACCAGGGTATCTAATCCTGTTTGCTCCCCACGCTTTCGCGCCTC
GZIPSVE01CRUN5     GTTTACGGCGTGGACTACCAGGGTATCTAATCCTGTTTGCTCCCCACGCTTTCGCGCCTC
                   ************************************************************

cluster            AGCGTCAGTATTCAGCCAGAAAGTCGCCTTCGCCACCGGTATCCCTCCCGATATCTACGA
GZIPSVE02HJ69D     AGCGTCAGTATTCAGCCAGAAAGTCGCCTTCGCCACCGGTATCCCTCCCGATATCTACGA
GZIPSVE01C6P61     AGCGTCAGTATTCAGCCAGAAAGTCGCCTTCGCCACCGGTATTCCTCCCGATATCTACGA
GZIPSVE01CRUN5     AGCGTCAGTATTCAGCCAGAAAGTCGCCTTCGCCACCGGTATTCCTCCCGATATCTACGA
                   ****************************************** *****************

cluster            ATTTCACCTCTACACCGGGAATTCCACTTTCCCCTCTGATACTCAAGCCGGGCAGTTTCA
GZIPSVE02HJ69D     ATTTCACCTCTACACCGGGAATTCCACTTTCCCCTCTGATACTCAAGCCGGGCAGTTTCA
GZIPSVE01C6P61     ATTTCACCTCTACACCGGGAATTCCACTTTCCCCTCTGATACTCAAGCCGGGCAGTTTCA
GZIPSVE01CRUN5     ATTTCACCTCTACACCGGGAATTCCACTTTCCCCTCTGATACTCAAGCCGGGCAGTTTCA
                   ************************************************************

cluster            AATGCACTTCCACGGTTAAGCCGTGGGCTTTCACATCTGACTTGCCAGGCCGCCTGCGCG
GZIPSVE02HJ69D     AATGCACTTCCACGGTTAAGCCGTGGGCTTTCACATCTGACTTGCCAGGCCGCCTGCGCG
GZIPSVE01C6P61     AATGCACTTCCACGGTTAAGCCGTGGGCTTTCACATCTGACTTGCCAGGCCGCCTGCGCG
GZIPSVE01CRUN5     AATGCACTTCCACGGTTAAGCCGTGGGCTTTCACATCTGACTTGCCAGGCCGCCTGCGCG
                   ************************************************************

cluster            CCCTTTACGCCCAGTGATTCCGAAC-----------------------------------
GZIPSVE02HJ69D     CCCTTTACGCCCAGTGATTCCGAAC-----------------------------------
GZIPSVE01C6P61     CCCTTTACGCCCAGTGATTCCGAACAACCGCTTGCACCCTCCGTATTACCGCGGCTGCTG
GZIPSVE01CRUN5     CCCTTTACGCCCAGTGATTCCGAACAAC-GCTTGCACCCTCCGTATTACCGCGGCTGCTG
                   *************************

cluster            -----------
GZIPSVE02HJ69D     -----------
GZIPSVE01C6P61     GCACGGAGTT-
GZIPSVE01CRUN5     GCACAGAGTTA
```
