# Supplementary material for: Assessing the Consequences of Denoising Marker-Based Metagenomic Data
Source: PLoS One. 2013 Mar 25;8(3):e60458. doi: 10.1371/journal.pone.0060458 (PMC3607570; doi:10.1371/journal.pone.0060458)
Supplement: File S8 — Alignment of a cluster of three reads formed by SeqNoise. The three reads differ in three positions, at each of which two of the reads agree. None of the three reads matches the dominant base at all three positions. Therefore, forming a true consensus requires a sequence that matches none of the three. (PDF) [file pone.0060458.s008.pdf]

```
cluster            AGTCGTACACTCCTACGGGAGGCAGCAGTGGGGAATATTGGACAATGGGCGAAAGCCTGA
GZIPSVE02G0323     AGTCGTACACTCCTACGGGAGGCAGCAGTGGGGAATATTGGACAATGGGCGAAAGCCTGA
GZIPSVE02HSRLA     AGTCGTACACTCCTACGGGAGGCAGCAGTGGGGAATATTGGACAATGGGCGAAAGCCTGA
GZIPSVE02IH7MV     AGTCGTACACTCCTACGGGAGGCAGCAGTGGGGAATATTGGACAATGGGCGAAAGCCTGA
                   ************************************************************

cluster            TCCAGCAATACCGCGTGAATGATGAAGGCCCTAGGGTTGTAAAGTTCTTTTAATAGGGAA
GZIPSVE02G0323     TCCAGCAATACCGCGTGAATGATGAAGGCCCTAGGGTTGTAAAGTTCTTTTAATAGGGAA
GZIPSVE02HSRLA     TCCAGCAATACCGCGTGAATGATGGAGGCCCTAGGGTTGTAAAGTTCTTTTAATAGGGAA
GZIPSVE02IH7MV     TCCAGCAATACCGCGTGAATGATGAAGGCCCTAGGGTTGTAAAGTTCTTTTAATAGGGAA
                   ************************ ***********************************

cluster            GATAATGACGGTACCTATAGAAAAAGCCCCGGCTAACTCCGTGCCAGCAGCCGCGGTAAG
GZIPSVE02G0323     GATAATGACGGTACCTATAGAAAAAGCCCCGGCTAACTCCGTGCCAGCAGCCGCGGTAAG
GZIPSVE02HSRLA     GATAATGACGGTACCTATAGAAAAAGCCCCGGCTAACTCCGTGCCAGCAGCCGCGGTAAG
GZIPSVE02IH7MV     GATAATGACGGTACCTATAGAAAAAGCCCCGGCTAACTCCGTGCCAGCAGCCGCGGTAAG
                   ************************************************************

cluster            ACGGAGGGGGCTAGCGTTATTCGGAATTACTGGGCGTAAAGGGCGCGTAGGCGGATTAGT
GZIPSVE02G0323     ACGGAGGGGGCTAGCGTTATTCGGAATTACTGGGCGTAAAGGGCGCGTAGGCGGATTAGT
GZIPSVE02HSRLA     ACGGAGGGGGCTAGCGTTATTCGGAATTACTGGGCGTAAAGGGCGCGTAGGCGGATTAGT
GZIPSVE02IH7MV     ACGGAGGGGGCTAGCGTTATTCGGAATTACTGGGCGTAAAGGGCGCGTAGGCGGATTAGT
                   ************************************************************

cluster            AAGTTGGGAGTGAAAGCCCGGGGCTTAACCTCGGAACTGCTTTCAAAACTGCTAGTCTTG
GZIPSVE02G0323     AAGTTGGGAGTGAAAGCCCGGGGCTTAACCTCGGAACTGCTTTCAAAACTGCTAGTCTTG
GZIPSVE02HSRLA     AAGTTGGGAGTGAAAGCCCGGGGCTTAACCTCGGAACTGCTTTCAAAACTGCTAGTCTTG
GZIPSVE02IH7MV     AAGTTGGGAGTGAAAGCCCGAGGCTTAACCTCGGAACTGCTTTCAAAACTGCTAGTCTTG
                   ********************* **************************************

cluster            AGTGAAGTAGGGGGTGATGGAATTCCTAGTGTAGAGGTGAAATTCTTAGATATTAGGAGG
GZIPSVE02G0323     AGTGAAGTAGGGGGTGATGGAATTCCTAGTGTAGAGGTGAAATTCTTAGATATTAGGAGG
GZIPSVE02HSRLA     AGTGAAGTAGGGGGTGATGGAATTCCTAGTGTAGAGGTGAAATTCTTAGATATTAGGAGG
GZIPSVE02IH7MV     AGTGAAGTAGGGGGTGATGGAATTCCTAGTGTAGAGGTGAAATTCTTAGATATTAGGAGG
                   ************************************************************

cluster            AACACCGGTGGTGAAGGCGGTCACCTGGACTTCAACTGACGCTGAGGCGCGAAAGCGTGG
GZIPSVE02G0323     AACACCGGTGGTGAAGGCGGTCACCTGGACTTCAACTGACGCTGAGGCGCGAAAGCGTGG
GZIPSVE02HSRLA     AACACCGGTGGCGAAGGCGGTCACCTGGACTTCAACTGACGCTGAGGCGCGAAAGCGTGG
GZIPSVE02IH7MV     AACACCGGTGGCGAAGGCGGTCACCTGGACTTCAACTGACGCTGAGGCGCGAAAGCGTGG
                   *********** ************************************************

cluster            GGAGCAAA
GZIPSVE02G0323     GGAGCAAA
GZIPSVE02HSRLA     GGAGCAAA
GZIPSVE02IH7MV     GGAGCAAA
                   ********
```
