# Supplementary material for: Assessing the Consequences of Denoising Marker-Based Metagenomic Data
Source: PLoS One. 2013 Mar 25;8(3):e60458. doi: 10.1371/journal.pone.0060458 (PMC3607570; doi:10.1371/journal.pone.0060458)
Supplement: File S9 — Results of other pipelines’ Filtering steps. A: CleanOpt. B: split_libraries (QIIME). C: trim.flows (mothur). (PDF) [file pone.0060458.s009.pdf]

A. CleanOpt

	Too few flows (< 360)	No signal for three flows	Large flow value (> 6.49)	Flow value between 0.50 and 0.70	Too many flows (> 720)	Total number
With truncations						
Reads eliminated	3,101	113	184	5,505	N/A	8,903
Reads truncated	N/A	677	40	17,184	7,080	24,981
Without truncations						
Reads eliminated	3,101	0	0	0	N/A	3,101
Reads truncated	N/A	0	0	0	0	0

40,596 flowgrams were retrieved by SplitKeys and analyzed by CleanOpt.pl.

B. split_libraries (QIIME)

	Minimum length (150 bp)	Maximum length (550 bp)	Sliding window of quality scores	Total number
Reads eliminated	1,277	182	2,220	3,679
Reads truncated	N/A	N/A	20,493	20,493

split_libraries.py was applied to our reads using the recommended (-l 150, -L 550, -w 50) and other default parameters.

C. trim.flows (mothur)

	Too few flows (< 449)	No signal for four flows	Flow value between 0.50 and 0.70	Too many flows (> 450)	Total number
Reads eliminated	4,794	193	10,490	N/A	15,477
Reads truncated	N/A	0	0	25,119	25,119

40,596 flowgrams were retrieved and analyzed by trim.flows using the default settings.
