# Supplementary material for: Assessing the Consequences of Denoising Marker-Based Metagenomic Data
Source: PLoS One. 2013 Mar 25;8(3):e60458. doi: 10.1371/journal.pone.0060458 (PMC3607570; doi:10.1371/journal.pone.0060458)
Supplement: File S12 — Alignment of two reads in SLP. The needledist algorithm of Esprit, based on quickdist, considers insertions and deletions of any size as being a single event. Therefore, it calculates the distance between these two reads as 1 mismatch / (212 matches + 1 mismatch) = 1/213 = 0.004695. These reads will be clustered by SLP if the cluster width parameter, -w, is greater than this distance. (PDF) [file pone.0060458.s012.pdf]

```
GZIPSVE02FSVFT      TAACCTTGCGGCCGTACTCCCCAGGCGGTCAACTTAATGCGTTAGCTGC-----------
GZIPSVE01DR8QC      TAACCTTGCGGCCGTACTCCCCAGGCGGTCAACTTAATGCGTTAGCTGCGCCACTAAGAG
                    *************************************************

GZIPSVE02FSVFT      ------------------------------------------------------------
GZIPSVE01DR8QC      CTCAAGGCTCCCAACGGCTAGTTGACATCGTTTACGGCGTGGACTACCAGGGTATCTAAT


GZIPSVE02FSVFT      ------------------------------------------------------------
GZIPSVE01DR8QC      CCTGTTTGCTCCCCACGCTTTCGCACCTCAGTGTCAGTATCAGTCCAGGTGGTCGCCTTC


GZIPSVE02FSVFT      ------------------------------------------------------------
GZIPSVE01DR8QC      GCCACTGGTGTTCCTTCCTATATCTACGCATCTCACCGCTACACAGGAAATTCCACCACC


GZIPSVE02FSVFT      -----------------------GTTTTGAATGCAGTTCCCAGGTTGAGCCCGGGGATTT
GZIPSVE01DR8QC      CTCTACCATACTCTAGTCAGTCAGTTTTGAATGCAGTTCCCAGGTTGAGCCCGGGGATTT
                                           *************************************

GZIPSVE02FSVFT      CACATCCAACTTAACTAACCACCTACGCGCGCTTTACGCCCAGTAATTCCGATTAACGCT
GZIPSVE01DR8QC      CACATCCAACTTAACTAACCACCTACGCGCGCTTTACGCCCAGTAATTCCGATTAACGCT
                    ************************************************************

GZIPSVE02FSVFT      TGCACCCCCTGTATTACCGCGGCTGCTGGCACAGAGTTAGCCGGTGCTTATTCTGTCGGT
GZIPSVE01DR8QC      TGCACCCCCTGTATTACCGCGGCTGCTGGCACAGAGTTAGCCGGTGCTTATTCTGTCGGT
                    ************************************************************

GZIPSVE02FSVFT      AACGTCAAAACAGCAAAGTATTAATTTACTGCCCTTCCTCCCAACTTAAAGTGCTTTACA
GZIPSVE01DR8QC      AACGTC------------------------------------------------------
                    ******

GZIPSVE02FSVFT      ATCCGAAGACCTTCTTCACACACGCGGCATGGCTGGATCAGGCTTTCGCCACTGTCCAAT
GZIPSVE01DR8QC      ------------------------------------------------------------


GZIPSVE02FSVFT      ATTCCCCA
GZIPSVE01DR8QC      --------
```
