# Supplementary material for: Assessing the Consequences of Denoising Marker-Based Metagenomic Data
Source: PLoS One. 2013 Mar 25;8(3):e60458. doi: 10.1371/journal.pone.0060458 (PMC3607570; doi:10.1371/journal.pone.0060458)
Supplement: File S13 — Pairwise alignment of three reads clustered by SLP. A: A chain of reads that are clustered together by SLP. B: The distance between the first and last reads far exceeds the cluster width of 0.005, because of the chaining effect. (PDF) [file pone.0060458.s013.pdf]

A

MID15_407 → MID15_2420 → MID15_6180 → MID15_924 → MID15_3210 → MID15_6325 → MID15_6323

B

```
MID15_6323      TGGGGAATTTTTCCGCAATGGGCGAAAAGCCTGACGGAGCAACGCCGCGTGAGGGATGAA
MID15_407       TGGGGAATTTT-CCGCAATGGGCGAAA-GCCTGACGGAGCAACGCCGCGTGAGGGATGAA
                *********** *************** ********************************

MID15_6323      GGCCTCTGGGCTGTAAACCTCTTTTTATCAAGGAAGAAGATCTGACGGTACTTGATGAAT
MID15_407       GGCCTCTGGGCTGTAAACCTCTTTT-ATCAAGGAAGAAGATCTGACGGTACTTGATGAAT
                ************************* **********************************

MID15_6323      AAGCCACGGCTAATTCCGTGCCAGCAGCCGCGGTAATACGGGAGTGGCAAGCGTTATCCG
MID15_407       AAGCCACGGCTAATTCCGTGCCAGCAGCCGCGGTAATACGGGAGTGGCAAGCGTTATCCG
                ************************************************************

MID15_6323      GAATTATTGGGCGTAAAGCGTCCGCAGGCGGTCT-GTCAAGTCTGCTGTTAAAGCGTG-A
MID15_407       GAATTATTGGGCGTAAAGCGTCCGCAGGCGGTCTTGT-AAGTCTGTTGTTAAAGCGTGGA
                ********************************** ** ******* ************ *

MID15_6323      GCTTAACTCCATTTCGGCAGTGGAAACTG-ACAGACTAGAGTGTGGTA------------
MID15_407       GCTTAACTCCATTTCAGCAATGGAAACTGTA-AGACTAGAGTGTGGTAGGGGCAGAGGGA
                *************** *** ********* * ****************

MID15_6323      ------------------------------------------------------------
MID15_407       ATTCCCGGTGTAGCGGTGAAATGCGTAGATATCGGGAAGAACACCAGTGGCGAAGGCGCT

MID15_6323      -----------------------------------
MID15_407       CTGCTGGGCCATAACTGACGCTCATGGACGAAAGCC
```
