# Supplementary material for: Assessing the Consequences of Denoising Marker-Based Metagenomic Data
Source: PLoS One. 2013 Mar 25;8(3):e60458. doi: 10.1371/journal.pone.0060458 (PMC3607570; doi:10.1371/journal.pone.0060458)
Supplement: File S14 — Pairwise alignments of three reads clustered by SLP. A, B: Pairs of reads that are identical over the shorter read’s length. When using a width of 0, the first and third reads are clustered together via the second read, despite their nonzero distance (C). (PDF) [file pone.0060458.s014.pdf]

A

```
MID12_200     TGGGGAATATTGGACAATGGGCGAAAGCCTGATCCAGCCATGCCGCGTGTGTGAAGAAGG
MID12_2955    TGGGGAATATTGGACAATGGGCGAAAGCCTGATCCAGCCATGCCGCGTGTGTGAAGAAGG
              ************************************************************

MID12_200     TCTTCGGATTGTAAAGCACTTTAAGTTGGGAGGAAGGGCAGTAAATTAATACTTTGCTGT
MID12_2955    TCTTCGGATTGTAAAGCACTTTAAGTTGGGAGGAAGGGCAGTAAATTAATACTTTGCTGT
              ************************************************************

MID12_200     TTTGACGTTACCGACAGAATAAGCACCGGCTAACTCTGTGCCAGCAGCCGCGGTAATACA
MID12_2955    TTTGACGTTACCGACAGAATAAGCACCGGCTAACTCTGTGCCAGCAGCCGCGGTAATACA
              ************************************************************

MID12_200     GAGGGTGCAAGCGTTAATCGGAATTACTGGGCGTAAAGCGCGCGTAGGTGGTTAGTTAAG
MID12_2955    GAGGGT------------------------------------------------------
              ******

MID12_200     TTGGATGTGAAATCCCCGGGCTCAACCTGGGAACTGCATTCAAAACTGACTGACTAGAGT
MID12_2955    ------------------------------------------------------------

MID12_200     ATGGTAGAGGGTGGTGGAATTTCCTGTGTAGCGGTGAAATGCGTAGATATAGGAAGGAAC
MID12_2955    ------------------------------------------------------------

MID12_200     ACCAGTGGCGAAGGCGACCACCTGGACTGATACTGACACTGAGGTG
MID12_2955    ----------------------------------------------
```

B

```
MID12_2955    TGGGGAATATTGGACAATGGGCGAAAGCCTGATCCAGCCATGCCGCGTGTGTGAAGAAGG
MID12_4567    TGGGGAATATTGGACAATGGGCGAAAGCCTGATCCAGCCATGCCGCGTGTGTGAAGAAGG
              ************************************************************

MID12_2955    TCTTCGGATTGTAAAGCACTTTAAGTTGGGAGGAAGGGCAGTAAATTAATACTTTGCTGT
MID12_4567    TCTTCGGATTGTAAAGCACTTTAAGTTGGGAGGAAGGGCAGTAAATTAATACTTTGCTGT
              ************************************************************

MID12_2955    TTTGACGTTACCGACAGAATAAGCACCGGCTAACTCTGTGCCAGCAGCCGCGGTAATACA
MID12_4567    TTTGACGTTACCGACAGAATAAGCACCGGCTAACTCTGTGCCAGCAGCCGCGGTAATACA
              ************************************************************

MID12_2955    GAGGGT------------------------------------------------------
MID12_4567    GAGGGTGCAAGCGTTACTCGGAATTACTGGGCGTAAAGCGTGCGTAGGTGGTCGTTTAAG
              ******

MID12_2955    ------------------------------------------------------------
MID12_4567    TCTGTTGTGAAAGCCCTGGGCTCAACCTGGGAACTGCAGTGGAAACTGGACGACTAGAGT

MID12_2955    ------------------------------------------------------------
MID12_4567    GTGGTAGAGGGTAGCGGAATTCCTGGTGTAGCAGTGAAATGCGTAGAGATCAGGAGGAAC

MID12_2955    -------------------------------------------------------
MID12_4567    ATCCATGGCGAAGGCAGCTACCTGGACCAACACTGACACTGAGGCACGAAAGCGT
```

C

```
MID12_200     TGGGGAATATTGGACAATGGGCGAAAGCCTGATCCAGCCATGCCGCGTGTGTGAAGAAGG
MID12_4567    TGGGGAATATTGGACAATGGGCGAAAGCCTGATCCAGCCATGCCGCGTGTGTGAAGAAGG
              ************************************************************

MID12_200     TCTTCGGATTGTAAAGCACTTTAAGTTGGGAGGAAGGGCAGTAAATTAATACTTTGCTGT
MID12_4567    TCTTCGGATTGTAAAGCACTTTAAGTTGGGAGGAAGGGCAGTAAATTAATACTTTGCTGT
              ************************************************************

MID12_200     TTTGACGTTACCGACAGAATAAGCACCGGCTAACTCTGTGCCAGCAGCCGCGGTAATACA
MID12_4567    TTTGACGTTACCGACAGAATAAGCACCGGCTAACTCTGTGCCAGCAGCCGCGGTAATACA
              ************************************************************

MID12_200     GAGGGTGCAAGCGTTAATCGGAATTACTGGGCGTAAAGCGCGCGTAGGTGGTTAGTTAAG
MID12_4567    GAGGGTGCAAGCGTTACTCGGAATTACTGGGCGTAAAGCGTGCGTAGGTGGTCGTTTAAG
              **************** *********************** ***********   *****

MID12_200     TTGGATGTGAAATCCCCGGGCTCAACCTGGGAACTGCATTCAAAACTG-ACTGACTAGAG
MID12_4567    TCTGTTGTGAAAGCCCTGGGCTCAACCTGGGAACTGCAGTGGAAACTGGAC-GACTAGAG
              *  * ******* *** ********************* *  ****** ** ********

MID12_200     TATGGTAGAGGGTGGTGGAATTTCCTG-TGTAGCGGTGAAATGCGTAGATAT-AGGAAGG
MID12_4567    TGTGGTAGAGGGTAGCGGAATT-CCTGGTGTAGCAGTGAAATGCGTAGAGATCAGGA-GG
              * *********** * ****** **** ****** ************** ** **** **

MID12_200     AACA-CCAGTGGCGAAGGCGACCACCTGGACTGATACTGACACTGAGGTG---------
MID12_4567    AACATCCA-TGGCGAAGGCAGCTACCTGGACCAACACTGACACTGAGGCACGAAAGCGT
              **** *** **********  * ********  * *************
```
