# Supplementary material for: Assessing the Consequences of Denoising Marker-Based Metagenomic Data
Source: PLoS One. 2013 Mar 25;8(3):e60458. doi: 10.1371/journal.pone.0060458 (PMC3607570; doi:10.1371/journal.pone.0060458)
Supplement: File S15 — Alignment of two reads clustered by Acacia. At two positions where the reads disagree, Acacia caused a deletion in both, instead of creating a substitution in one of the them. The reason for the deletion of the C near the 3′ end of the read “FH6HB” is unknown. (PDF) [file pone.0060458.s015.pdf]

```
cluster           ATATCGCGAGCCTACGGGAGGCAGCAGTGGGGAATATTGGACAATGGGCGAAAGCC-GAT
GZIPSVE02FH6HB    ATATCGCGAGCCTACGGGAGGCAGCAGTGGGGAATATTGGACAATGGGCGAAAGCCCGAT
GZIPSVE02IEKVK    ATATCGCGAGCCTACGGGAGGCAGCAGTGGGGAATATTGGACAATGGGCGAAAGCCTGAT
                  ******************************************************** ***

cluster           CCAGCCATGCCGCGTGTGTGAAGAAGGTCTTCGGATTGTAAAGCACTTTAAGTTGGGAGG
GZIPSVE02FH6HB    CCAGCCATGCCGCGTGTGTGAAGAAGGTCTTCGGATTGTAAAGCACTTTAAGTTGGGAGG
GZIPSVE02IEKVK    CCAGCCATGCCGCGTGTGTGAAGAAGGTCTTCGGATTGTAAAGCACTTTAAGTTGGGAGG
                  ************************************************************

cluster           AAGGGCAGTAAATTAATACTTTGCTGTTTTGACGTTACCGACAGAATAAGCACCGGCTAA
GZIPSVE02FH6HB    AAGGGCAGTAAATTAATACTTTGCTGTTTTGACGTTACCGACAGAATAAGCACCGGCTAA
GZIPSVE02IEKVK    AAGGGCAGTAAATTAATACTTTGCTGTTTTGACGTTACCGACAGAATAAGCACCGGCTAA
                  ************************************************************

cluster           CTCTGTGCCAGCAGCCGCGGTAATACAGAGGGTGCAAGCGTTAATCGGAATTACTGGGCG
GZIPSVE02FH6HB    CTCTGTGCCAGCAGCCGCGGTAATACAGAGGGTGCAAGCGTTAATCGGAATTACTGGGCG
GZIPSVE02IEKVK    CTCTGTGCCAGCAGCCGCGGTAATACAGAGGGTGCAAGCGTTAATCGGAATTACTGGGCG
                  ************************************************************

cluster           TAAAGCGCGCGTAGGTGGTTAGTTAAGTTGGATGTGAA-TCCCCGGGCTCAACCTGGGAA
GZIPSVE02FH6HB    TAAAGCGCGCGTAGGTGGTTAGTTAAGTTGGATGTGAAATCCCCGGGCTCAACCTGGGAA
GZIPSVE02IEKVK    TAAAGCGCGCGTAGGTGGTTAGTTAAGTTGGATGTGAAGTCCCCGGGCTCAACCTGGGAA
                  ************************************** *********************

cluster           CTGCATTCAAAACTGACTGACTAGAGTATGGTAGAGGGTGGTGGAATTTCCTGTGCAGCG
GZIPSVE02FH6HB    CTGCATTCAAAACTGACTGACTAGAGTATGGTAGAGGGTGGTGGAATTTCCTGTGCAGCG
GZIPSVE02IEKVK    CTGCATTCAAAACTGACTGACTAGAGTATGGTAGAGGGTGGTGGAATTTCCTGTGCAGCG
                  ************************************************************

cluster           GTGAAATGCGTAGATATAGGAAGGAACACCAGTGGCGAAGGCGACCAC-TGGACTGATAC
GZIPSVE02FH6HB    GTGAAATGCGTAGATATAGGAAGGAACACCAGTGGCGAAGGCGACCAC-TGGACTGATAC
GZIPSVE02IEKVK    GTGAAATGCGTAGATATAGGAAGGAACACCAGTGGCGAAGGCGACCACCTGGACTGATAC
                  ************************************************ ***********

cluster           TGACACTGAGGTGCGAAAGCGTGGGGAGCAAACAGGATTAGATACCCTGGTAGTCCACGC
GZIPSVE02FH6HB    TGACACTGAGGTGCGAAAGCGTGGGGAGCAAACAGGATTAGATACCCTGGTAGTCCACGC
GZIPSVE02IEKVK    TGACACTGAGGTGCGAAA------------------------------------------
                  ******************

cluster           CGTAAACGATGTCAA-TAGCCGTTGGGA
GZIPSVE02FH6HB    CGTAAACGATGTCAACTAGCCGTTGGGA
GZIPSVE02IEKVK    ----------------------------
```
