# Supplementary material for: Assessing the Consequences of Denoising Marker-Based Metagenomic Data
Source: PLoS One. 2013 Mar 25;8(3):e60458. doi: 10.1371/journal.pone.0060458 (PMC3607570; doi:10.1371/journal.pone.0060458)
Supplement: File S16 — Changes made at each step of the denoising pipelines to an independent dataset. (PDF) [file pone.0060458.s016.pdf]

	Reference reads (stage)	Query reads (stage)	Number of reads analyzed	Mean 3′ gap (S.D.)	Substitutions	Insertions	Deletions	Total changes
AmpliconNoise								
Filtering	0	1A	42,371	-38.2 (64.8)	68	663	457	1,188
PyroNoise	1A	2A	42,371	27.3 (56.2)	8,575	4,673	3,315	16,563
Accordion effect	0	2A	42,371	-10.4 (38.0)	18,004	13,025	35,514	66,543
Truncation	2A	3A	42,371	-15.5 (9.8)	0	0	0	0
SeqNoise	3A	4A	42,371	4.3 (27.0)	23,552	2,123	1,635	27,310
Perseus	4A	5A	41,583	0.0 (0.0)	0	0	0	0
Net results	0	5A	41,583	-21.2 (25.6)	40,794	13,397	34,647	88,838
CleanOpt (trunc)	0	1A	42,244	-38.8 (64.2)	0	0	340	340
PyroNoise	1A	2A	42,244	25.9 (55.6)	8,553	4,893	2,858	16,304
Accordion effect	0	2A	42,244	-12.3 (37.7)	17,680	12,545	34,528	64,753
CleanOpt (no trunc)	0	1A	46,307	0.0 (0.0)	186	73	2,302	2,561
PyroNoise	1A	2A	46,307	1.4 (9.9)	11,565	13,004	14,525	39,094
Accordion effect	0	2A	46,307	1.4 (9.9)	11,373	11,258	14,959	37,590
QIIME								
split_libraries	0	1B	44,918	-18.8 (49.2)	413	0	0	413
Pre-clustering	1B	2B	44,918	19.3 (49.4)	82	867	332	1,281
Accordion effect	0	2B	44,918	0.4 (5.0)	716	3,040	1,643	5,399
denoiser	2B	3B	44,918	1.2 (12.8)	161,485	46,383	69,031	276,899
ChimeraSlayer	3B	4B	44,627	0.0 (0.0)	0	0	0	0
Net results	0	4B	44,627	1.6 (13.7)	161,221	45,888	67,288	274,397
mothur								
trim.flows	0	1C	38,989	-155.7 (19.0)	4	169	92	265
shhh.flows	1C	2C	38,989	0.0 (0.1)	3,948	1,583	947	6,478
Accordion effect	0	2C	38,989	-155.7 (19.0)	4,031	1,541	1,185	6,757
trim.seqs	2C	3C	38,989	0.0 (0.0)	0	0	0	0
shhh.seqs	3C	4C	38,989	0.0 (1.2)	8,904	377	267	9,548
chimera.uchime	4C	5C	38,423	0.0 (0.0)	0	0	0	0
Net results	0	5C	38,423	-155.7 (19.1)	12,902	1,877	1,550	16,329
SLP								
split_libraries	0	1D	42,511	-23.5 (9.9)	0	0	0	0
slp.pl	1D	2D	42,511	-2.7 (12.2)	67,131	9,966*	16,244*	93,341
Net results	0	2D	42,511	-26.2 (10.3)	67,159	10,212*	16,705*	94,076
Acacia								
Net results	0	1E	45,184	-3.4 (19.8)	10,883	4,535	5,178	20,596

All data are in units of base pairs, except those indicated by * (units of events).
